# Plasma metabolomics exhibit response to therapy in chronic thromboembolic pulmonary hypertension

**DOI:** 10.1183/13993003.03201-2020

**Published:** 2021-04-01

**Authors:** Emilia M. Swietlik, Pavandeep Ghataorhe, Kasia I. Zalewska, John Wharton, Luke S. Howard, Dolores Taboada, John E. Cannon, UK National Cohort Study of PAH, Nicholas W. Morrell, Martin R. Wilkins, Mark Toshner, Joanna Pepke-Zaba, Christopher J. Rhodes

**Affiliations:** 1 Dept of Medicine, University of Cambridge, Cambridge, UK; 2 National Pulmonary Hypertension Service, Royal Papworth Hospital, Cambridge, UK; 3 National Heart and Lung Institute, Medicine, Imperial College London, London, UK; 4 Respiratory Unit, University Hospital Llandough, Cardiff, UK; 5 National Pulmonary Hypertension Service, Imperial College Healthcare NHS Trust and NHLI, Imperial College, Hammersmith Hospital, London, UK

## Abstract

Pulmonary hypertension is a condition with limited effective treatment options. Chronic thromboembolic pulmonary hypertension (CTEPH) is a notable exception, with pulmonary endarterectomy (PEA) often proving curative. This study investigated the plasma metabolome of CTEPH patients, estimated reversibility to an effective treatment and explored the source of metabolic perturbations.

We performed untargeted analysis of plasma metabolites in CTEPH patients compared to healthy controls and disease comparators. Changes in metabolic profile were evaluated in response to PEA. A subset of patients were sampled at three anatomical locations and plasma metabolite gradients calculated.

We defined and validated altered plasma metabolite profiles in patients with CTEPH. 12 metabolites were confirmed by receiver operating characteristic analysis to distinguish CTEPH and both healthy (area under the curve (AUC) 0.64–0.94, all p<2×10^−5^) and disease controls (AUC 0.58–0.77, all p<0.05). Many of the metabolic changes were notably similar to those observed in idiopathic pulmonary arterial hypertension (IPAH). Only five metabolites (5-methylthioadenosine, N1-methyladenosine, N1-methylinosine, 7-methylguanine, N-formylmethionine) distinguished CTEPH from chronic thromboembolic disease or IPAH. Significant corrections (15–100% of perturbation) in response to PEA were observed in some, but not all metabolites. Anatomical sampling identified 188 plasma metabolites, with significant gradients in tryptophan, sphingomyelin, methionine and Krebs cycle metabolites. In addition, metabolites associated with CTEPH and gradients showed significant associations with clinical measures of disease severity.

We identified a specific metabolic profile that distinguishes CTEPH from controls and disease comparators, despite the observation that most metabolic changes were common to both CTEPH and IPAH patients. Plasma metabolite gradients implicate cardiopulmonary tissue metabolism of metabolites associated with pulmonary hypertension and metabolites that respond to PEA surgery could be a suitable noninvasive marker for evaluating future targeted therapeutic interventions.

## Abstract 2

**Metabolic profiles can distinguish CTEPH from controls and disease comparators, but most metabolic changes are common to both CTEPH and IPAH. Cardiopulmonary tissue metabolism is relevant to PH and metabolites do respond to PEA surgery.** https://bit.ly/368ogtG

## Introduction

Pulmonary hypertension (PH) is defined by persistent elevation of resting mean pulmonary artery pressure and is associated with an increased risk of right heart failure and premature death [1]. Progress in medical therapies for PH has been limited to pulmonary arterial hypertension (PAH) and chronic thromboembolic pulmonary hypertension (CTEPH). Moreover, this has not been related to discovery of new disease mechanisms, but to improvements in targeting known pathways responsible for vasodilation and strategies related to early combination and escalation of treatments. CTEPH remains the only class of PH for which a potential cure exists, by means of a pulmonary endarterectomy (PEA) which commonly normalises haemodynamics [2, 3]. This provides an invaluable opportunity to study pathobiology and response to treatment [4].

Metabolomics allows high-dimensional molecular mapping of disease presentations and the potential to define endophenotypes. We and others have previously reported the plasma metabolomic profiles of patients with idiopathic and heritable pulmonary arterial hypertension (IPAH/HPAH) [5]. Here we compare the plasma metabolomic profiles of patients with CTEPH with those of other disease and healthy controls and patients with IPAH/HPAH and seek to establish whether metabolic alterations are corrected by PEA. In addition, we use plasma metabolome gradients between superior vena cava (SVC), pulmonary artery (PA) and radial artery (ART) to investigate the tissue of origin of any perturbation.

## Methods

### Study participants and sample collection

**FIGURE 1:**
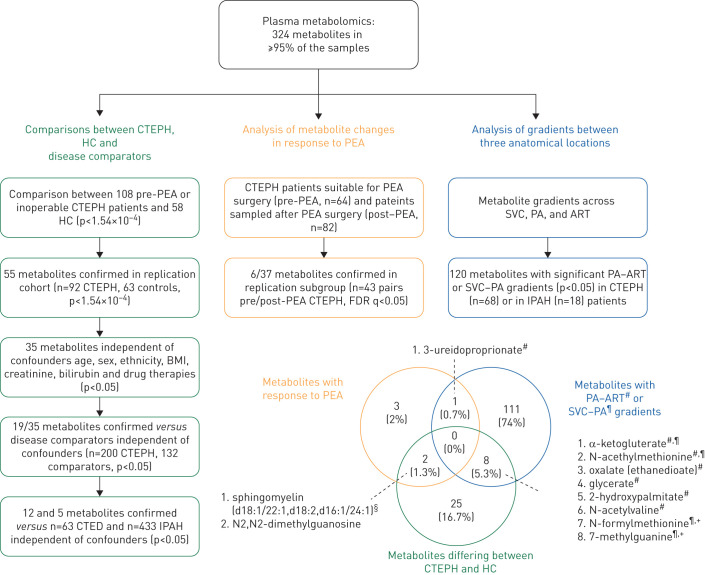
Main analyses study flowchart and overlap of metabolites identified in the main analyses. Individuals analysed consisted of patients with chronic thromboembolic pulmonary hypertension (CTEPH), healthy controls (HC), disease comparators (DC; referrals found not to have pulmonary hypertension (PH) or those with chronic thromboembolic disease (CTED) but not PH) and patients with idiopathic (IPAH) or hereditary pulmonary arterial hypertension (HPAH). Patients with inoperable CTEPH or those sampled before pulmonary endarterectomy (PEA) were used in the main analyses. Additionally, 43 patients were sampled after PEA, while a further 82 patients consented for sampling only post-PEA. Venn diagram depicts overlap in metabolites identified by comparisons of CTEPH compared to healthy controls, CTEPH patients analysed before and after PEA surgery, and plasma gradients across tissue vascular beds relevant to PH, specifically the pulmonary artery (PA) to radial artery (ART) and superior vena cava (SVC) to PA gradients. BMI: body mass index; FDR: false discovery rate. ^#^: PA–ART gradient; ^¶^: SVC–PA gradient; ^+^: metabolites which also differed in the analysis of CTEPH against IPAH patients; ^§^: probable metabolite identity, but unconfirmed (see methods).

Patients attending the National Pulmonary Hypertension Service at Hammersmith Hospital (London, UK) and Royal Papworth Hospital (Cambridge, UK) donated blood samples with informed consent and approval of local research ethics committees (reference numbers 17/LO/0563 and 15/EE/0201). Total samples collected in the main cohorts and analysis plan are detailed in table 1 and figure 1, respectively.

**TABLE 1:** Main cohort characteristics

	**HC**	**DC**	**CTED**	**IPAH/HPAH**	**CTEPH-discovery**	**CTEPH-replication**	**Patients sampled at three locations**
**Subjects**	121	132	63	433	108	92	86
**Demographics**							
Age at sampling years	51 (37–57)	59 (43–69)	60 (45–70)	54 (41–67)	68 (56–76)	66 (53–77)	64 (50–71)
Female	78 (64)	90 (68)	27 (43)	305 (70)	41 (38)	56 (61)	42 (49)
Ethnicity: European	66 (55)	57 (43)	33 (66)	358 (83)	82 (76)	68 (74)	74 (86)
BMI kg·m^−2^	25 (24–30)	27 (24–30)	30 (26–34)	28 (24–32)	27 (24–30)	28 (25–32)	29 (25–34)
WHO functional class							
I	ND	ND	4 (17)	28 (7)	2 (2)	7 (9)	5 (6)
II	ND	ND	12 (52)	105 (26)	23 (22)	13 (16)	30 (35)
III	ND	ND	7 (30)	238 (58)	73 (69)	53 (67)	48 (56)
IV	ND	ND	0 (0)	38 (9)	8 (8)	6 (8)	3 (3)
6-min walk distance m	ND	ND	387 (312–452)	336 (187–420)	282 (146–384)	218 (96–352)	352 (260–436)
Creatinine mmol·L^−1^	ND	71 (63–89)	78 (72–86)	83 (69–104)	84 (70–106)	88 (74–103)	88 (72–103)
Bilirubin μmol·L^−1^	ND	9 (7–14)	9 (7–13)	11 (8–17)	12 (9–19)	12 (9–20)	11 (8–14)
Albumin g·L^−1^	ND	40 (38–42)	40 (39–42)	40 (37–44)	38 (35–40)	38 (36–40)	38 (36–40)
CRP mg·L^−1^	ND	3 (1–6)	2 (1–3)	4 (2–7)	3 (2–7)	5 (3–11)	2 (1–7)
**Haemodynamics at diagnosis**							
mRAP mmHg	ND	6 (4–9)	6 (4–8)	9 (6–13)	9 (6–12)	9 (6–13)	8 (5–11)
mPAP mmHg	ND	20 (16–23)	20 (17–22)	53 (44–62)	41 (33–54)	45 (34–54)	38 (33–44)
mPAWP mmHg	ND	11 (9–14)	10 (8–13)	10 (7–13)	12 (9–14)	11 (8–13)	10 (8–13)
PVR WU	ND	1.7 (1.1–2.5)	1.8 (1.2–2.3)	11.1 (6.8–15.7)	8.0 (4.9–11.3)	7.9 (4.8–11.6)	5.5 (4.0–8.1)
Cardiac output L·min^−1^	ND	4.8 (3.7–5.9)	5.2 (4.5–6.0)	3.8 (3.0–4.8)	3.9 (3.3–4.6)	4.0 (3.0–4.8)	4.6 (4.1–5.7)
**Comorbidities and medication**							
COPD	0 (0)	20 (15)	4 (6)	65 (15)	8 (7)	12 (13)	8 (9)
Diabetes	0 (0)	15 (11)	5 (8)	82 (19)	11 (10)	8 (9)	9 (10)
Atherosclerosis	0 (0)	14 (11)	2 (4)	59 (14)	32 (37)	17 (18)	20 (23)
Atrial arrhythmia	0 (0)	22 (17)	3 (5)	57 (13)	20 (19)	22 (24)	8 (9)
Hypertension	0 (0)	39 (30)	12 (19)	103 (24)	36 (33)	26 (28)	29 (34)
Dyslipidaemia	0 (0)	16 (12)	13 (21)	44 (10)	22 (20)	14 (15)	25 (29)
PDE-5i	0 (0)	0 (0)	1 (2)	283 (65)	40 (39)	34 (37)	27 (31)
ERA	0 (0)	0 (0)	0 (0)	232 (54)	28 (27)	21 (23)	15 (17)
Riociguat	0 (0)	0 (0)	0 (0)	0 (0)	0 (0)	0 (0)	3 (3)
Prostanoid	0 (0)	0 (0)	0 (0)	78 (18)	2 (2)	1 (1)	4 (5)
Anticoagulation	0 (0)	44 (33)	27 (84)	291 (67)	104 (96)	87 (95)	Stopped for RHC
Loop diuretic	0 (0)	24 (18)	5 (8)	228 (53)	47 (44)	46 (50)	46 (53)
Potassium-sparing diuretic	0 (0)	6 (5)	2 (3)	104 (24)	24 (22)	26 (28)	17 (20)
Statin	0 (0)	40 (30)	11 (22)	112 (26)	38 (42)	35 (38)	25 (29)
CCB	0 (0)	22 (17)	5 (8)	75 (17)	4 (4)	9 (10)	12 (14)
Digoxin	0 (0)	11 (8)	1 (2)	68 (16)	7 (6)	8 (9)	3 (3)
Antidiabetic drugs	0 (0)	13 (10)	4 (9)	62 (14)	11 (13)	8 (9)	9 (10)
Iron supplementation	0 (0)	7 (5)	1 (2)	50 (12)	8 (10)	6 (7)	7 (8)
ACEi	0 (0)	44 (33)	11 (17)	100 (23)	30 (28)	26 (28)	12 (14)

Data are presented as n, median (interquartile range) or n (%), unless otherwise stated. Ethnicity is shown for subjects who self-declared. A further group of 82 CTEPH patients sampled only after pulmonary endartectomy surgery are detailed in supplementary table S1. HC: healthy controls; DC: disease controls; CTED: chronic thromboembolic disease; IPAH/HPAH: idiopathic and heritable pulmonary arterial hypertension; CTEPH: chronic thromboembolic pulmonary hypertension; BMI: body mass index; WHO: World Health Organization; CRP: C-reactive protein; mRAP: mean right atrial pressure; mPAP: mean pulmonary artery pressure; mPAWP: mean pulmonary artery wedge pressure; PVR: pulmonary vascular resistance; WU: Wood unit; PDE-5i: phosphodiesterase-5 inhibitor; ERA: endothelin receptor antagonist; CCB: calcium channel blocker; ACEi: angiotensin-converting enzyme inhibitor; ND: not determined; RHC: right-heart catheterisation.

Patients were recruited at Hammersmith Hospital (December 10, 2002 to May 20, 2019) and Papworth Hospital (September 30, 2015 to January 10, 2019) with diagnoses of CTEPH, IPAH or HPAH. Control samples were obtained from healthy volunteers, patients with chronic thromboembolic disease (CTED) [6] and disease control individuals; the latter presented as symptomatic patients who were subsequently found not to have pulmonary hypertension [5]. Additional IPAH/HPAH patients were included as a comparator group and sampled between February 19, 2014 and June 24, 2015 from other expert centres in the UK as part of the National Cohort Study of Idiopathic and Heritable Pulmonary Arterial Hypertension (ClinicalTrials.gov NCT01907295). Venous blood samples were drawn from the antecubital fossa in to EDTA Vacutainer tubes (BD, Oxford, UK), immediately inverted eight to 10 times, put on ice, centrifuged (1300×*g*, 15 min at 4°C) within 30 min, and plasma stored at −80°C until required.

Initially, a discovery cohort of 108 consecutive CTEPH patients was compared to 58 healthy controls and the results replicated in a second cohort of 92 CTEPH patients compared to a distinct healthy control group (n=63) (figure 1). Similar proportions were deemed operable for PEA surgery (59 out of 108 and 48 out of 92) in the two CTEPH cohorts. To understand the specificity of any differences for CTEPH, metabolite profiles were compared with disease control individuals (n=132), patients with CTED (n=63) and IPAH/HPAH (n=433) (table 1 and figure 1).

In the second arm of the study, we evaluated the metabolite profiles of CTEPH patients before and after PEA surgery (figure 1). We compared metabolite levels in CTEPH patients deemed suitable for PEA surgery (pre-PEA, n=64) with matched (based on clinical characteristics) patients sampled after PEA surgery (post-PEA, n=82; supplementary table S1), and then analysed differences in a separate group of 43 patients who were sampled both before and after PEA surgery. All post-PEA samples were obtained after full recovery from surgery, at median 37 months for unpaired and 5.8 months for paired samples, and both groups exhibited similar reductions in mean pulmonary artery pressures and pulmonary vascular resistance (supplementary table S1).

A further set of patients with diagnoses of CTEPH (n=68) or IPAH/HPAH (n=18) at Papworth Hospital were sampled during elective right heart catheterisation between 2015 and 2017, allowing simultaneous sample collection from the superior vena cava, proximal portion of pulmonary artery and radial artery and haemodynamic measurements. Exclusion criteria included left ventricular systolic and or diastolic dysfunction, significant valvular heart disease, chronic kidney disease stage 4 or 5, chronic liver disease, liver failure or alcohol abuse, current illicit substance use, active infection and peripheral arterial vascular disease. Patients were sampled between 09:30 h and 12:30 h.

### Metabolomic analysis

Metabolomic profiling by ultra-performance liquid chromatography mass spectrometry (LC-MS) was conducted on the Discovery HD4TM Global Metabolomics platform by Metabolon, Inc. (Durham, NC, USA) [7]; data were provided as semi-quantitative metabolite levels, annotated with pathways, as described previously [5]. Glycerophospholipid groups are abbreviated as follows. Glycerophosphorylcholine: GPC; glycerophosphoethanolamine: GPE; glycerophosphatidylinositol: GPI; glycerophosphatidylserine: GPS.

### Statistical analysis

We pre-processed metabolite data as described previously [5]. Briefly, metabolites were normalised by Box–Cox transformations [5] and samples where metabolites were undetected were imputed with the minimum detected level for the metabolite. Only 324 nonxenobiotic metabolites detected in ≥95% of samples were included. All data were z-score transformed based on healthy control data for ease of interpretation. In order to account for any between batch variability a quantile normalisation approach was utilised, which sets the distribution of metabolite levels in each sample to the average distribution of all samples, making them directly comparable [8]. Previously, this has been used in metabolomics LC-MS data to minimise experimental variation due to a variety of causes, including experiments being conducted at different times [8], using more than one instrument and different sample processing procedures [9].

Initial group comparisons between controls and patients were performed using nonparametric Mann–Whitney U-tests (as transformations did not eliminate skew). Comparisons before and after PEA surgery in paired samples was conducted using the Wilcoxon signed-rank test. Comparisons of demographic features between study groups were conducted using the Kruskal–Wallis (continuous data) or Chi-squared (categorical data) tests.

To assess the relationships between metabolite levels, diagnoses and potential confounders, regression models included preserved renal function defined as creatinine <75 µmol·L^−1^, and liver function as bilirubin <21 µmol·L^−1^ [5]. In the healthy control group, preserved renal and hepatic function was assumed as clinical assay data was unavailable.

Paired Wilcoxon signed-rank test was used for comparisons of metabolites abundance between sampling sites. False discovery rate correction was used to minimise false positive rate. Baseline clinical characteristics were expressed as numbers and percentages for categorical variables and mean±sd or median (interquartile range) for continuous variables according to data distribution. Comparisons of clinical characteristics between study groups were performed with parametric and nonparametric tests as per data distribution. Data were analysed and visualised using R (www.R-project.org/)

Pathway enrichment analysis on metabolites showing tissue gradients was performed with Fisher's exact test with all detected metabolites in each pathway as background. Undirected relevance network analysis [10] was performed to investigate the inter-relationship between metabolites that showed gradients across sampling sites; highly correlated metabolites (Spearman's ρ>0.9) were visualised using the *tidygraph* R package. Additionally, Spearman's correlation was performed to assess relationships between discriminatory metabolites and normalised clinically relevant (diagnostic or prognostic) variables. The results were visualised using *ggplot2*, *ggpubr*, *pROC*, *ggdendro* and *egg* R packages.

## Results

### Study participants

Baseline characteristics and laboratory data are shown in table 1 and supplementary table S1. Patients with PH show altered haemodynamics and impaired exercise capacity and an overview of the main comparison groups is given in figure 1 with details in table 1.

### Altered plasma metabolite profiles in CTEPH patients

First, we compared plasma metabolite levels in two sets of samples from pre-PEA or inoperable CTEPH patients and healthy control subjects (figure 1). Plasma levels of 55 metabolites distinguished CTEPH patients from healthy controls in both discovery and replication analyses following Bonferroni correction (mean differences to controls ranging −0.33– −1.53 sd and +0.84–2 sd, p<1.54×10^−4^; supplementary table S2). Of these, 35 metabolites distinguished CTEPH from healthy controls after correcting for potential confounders such as age, sex, ethnicity, body mass index, creatinine, bilirubin and drug therapies (p<0.05; supplementary table S2). Age affected 17 of these metabolites, but the average effect of CTEPH was ∼50–100 times greater; and sex affected 11 out of 35 metabolites, with the effect of CTEPH being 1.5–3.2 times the effect of sex (supplementary table S3 and supplementary figure S1).

**FIGURE 2:**
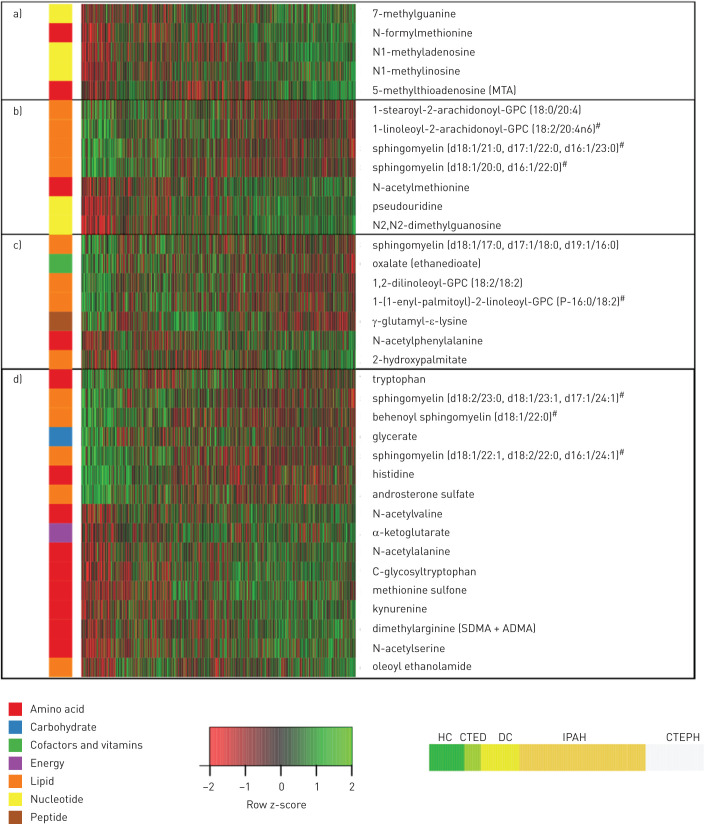
Heatmap of 35 metabolites that distinguish chronic thromboembolic pulmonary hypertension (CTEPH) patients from healthy controls (HC) and disease comparators (DC) independent of confounders. Metabolites a) distinguishing CTEPH from all other groups; b) distinguishing CTEPH from HC, DC and chronic thromboembolic disease (CTED); c) distinguishing CTEPH from HC and DC independent of confounders; and d) distinguishing CTEPH from HC independent of confounders. ^#^: probable metabolite identity, but unconfirmed (see methods).

**FIGURE 3:**
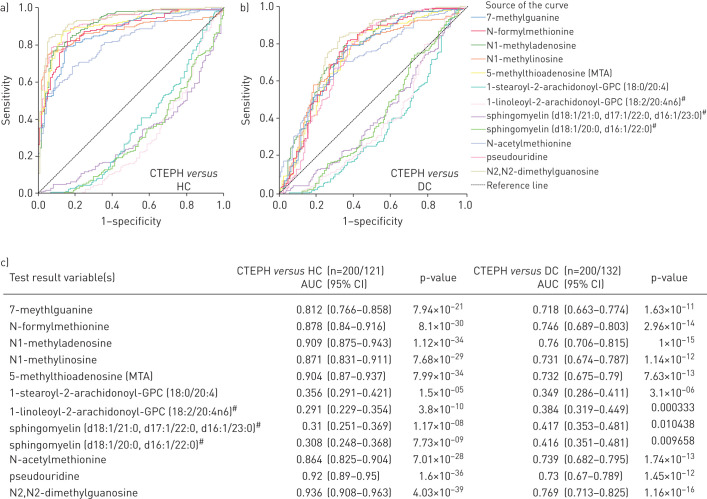
Receiver operating characteristic (ROC) analysis of key metabolites distinguishing chronic thromboembolic pulmonary hypertension (CTEPH) from healthy controls (HC) and disease controls (DC). ROC curves demonstrate ability of metabolites to distinguish CTEPH from a) HC and b) DC; c) areas under the curve (AUC) (95% CI). ^#^: probable metabolite identity, but unconfirmed (see methods).

**FIGURE 4:**
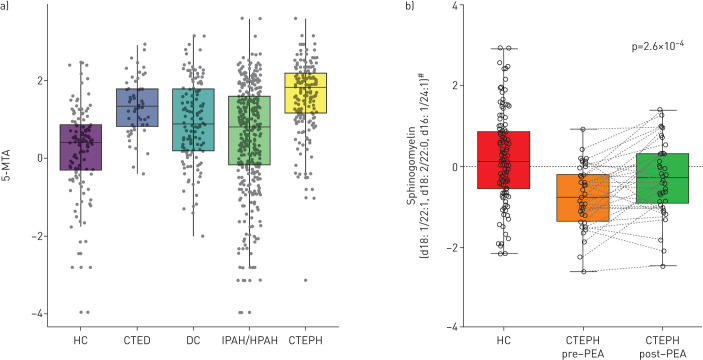
Box and dot-plots of plasma levels of key metabolites. a) 5-methylthioadenosine (MTA) in healthy controls (HC), chronic thromboembolic disease (CTED) patients without pulmonary hypertension, disease controls (DC), idiopathic or heritable pulmonary arterial hypertension (IPAH/HPAH) and chronic thromboembolic pulmonary hypertension (CTEPH). Levels in CTEPH are significantly different compared to HC (p=5.2×10^−5^) and DC (p=9.3×10^−4^) after correcting for confounders and *versus* CTED (p=1.9×10^−3^) and IPAH (p=3.8×10^−13^). b) Boxplot of sphingomyelin (d18:1/22:1, d18:0/22:0, d16:1/24:1) levels in paired plasma samples from 43 CTEPH patients, taken before and after pulmonary endarterectomy surgery, compared with HC. ^#^: probable metabolite identity, but unconfirmed (see methods).

Of the 35 discriminating metabolites, a subset of 19 also distinguished CTEPH patients from disease controls after correcting for potential confounders (p<0.05; figure 2); 10 were increased, including modified nucleosides (*e.g.* N2,N2-dimethylguanosine), monohydroxy-fatty acids and metabolites of polyamine and methionine metabolism; and nine, including phosphatidylcholines, oxalate, γ-glutamyl-ε-lysine and several sphingomyelins, were decreased (supplementary table S2). In addition, 12 metabolites were significantly different between CTEPH and CTED patients (p<0.05), the latter group being included as a control for underlying chronic thromboembolism without pulmonary hypertension and anticoagulation therapy (table 2, supplementary table S2). These 12 metabolites were confirmed by receiver operating characteristic analysis to distinguish CTEPH and both healthy (area under the curve (AUC) 0.64–0.94, all p<2×10^−5^) and disease controls (AUC 0.58–0.77, all p<0.05; figure 3). Sensitivities and specificities of the best cut-offs reached 57–92% and 35–94% (supplementary table S4). Finally, five metabolites significantly distinguished CTEPH from the PH comparator group of IPAH patients with the most marked difference being in 5-methylthioadenosine (figures 2 and 4a, table 2, supplementary table S2).

**TABLE 2:** Metabolites distinguishing chronic thromboembolic pulmonary hypertension (CTEPH) from healthy (HC) and disease controls (DC)

	**Metabolic pathway**	**Discovery**			**Replication**			**Linear regression with confounders p-value**		**Comparator groups**			
		**CTEPH**	**HC**	**p-value**	**CTEPH**	**HC**	**p-value**	**HC *versus* CTEPH**	**DC *versus* CTEPH**	**CTED**	**p-value**	**IPAH/HPAH**	**p-value**
**Significant in all analyses**													
5-MTA	Polyamine metabolism	1.72±0.84	0.07±1.22	**5.00×10^−17^**	1.55±0.91	0.32±1.12	**6.95×10^−12^**	**5.21×10^−05^**	**0.0009**	1.34±0.73	**0.0019**	0.86±1.26	**3.75×10^−13^**
N1-Methyladenosine	Purine metabolism, adenine-containing	1.58±0.78	0.03±1.01	**3.02×10^−17^**	1.67±0.61	0.4±1.1	**8.10×10^−14^**	**4.55×10^−05^**	**0.0105**	0.91±1.08	**2.07×10^−06^**	1.21±0.93	**1.56×10^−06^**
N1-Methylinosine	Purine metabolism, (hypo)xanthine/inosine-containing	1.64±1.54	0±1.1	**1.51×10^−12^**	1.91±1.45	0.41±1.06	**8.70×10^−13^**	**7.20×10^−05^**	**2.35×10^−05^**	1.59±1.11	**0.0406**	1.65±1.11	**0.008**
7-Methylguanine	Purine metabolism, guanine-containing	1.23±1.09	0.01±1.16	**8.47×10^−10^**	1.27±1.25	0.45±1.19	**0.0001**	**0.0007**	**0.0099**	0.54±1.08	**4.18×10^−05^**	0.95±1.28	**0.019**
N-Formylmethionine	Methionine, cysteine, SAM and taurine metabolism	1.45±0.88	0.05±1.02	**6.10×10^−14^**	1.51±0.78	0.36±1.11	**4.27×10^−11^**	**0.0024**	**0.0042**	1.22±0.78	**0.014**	1.31±0.86	**0.0406**
**Significant *versus* HC, DC and CTED**													
Sphingomyelin (d18:1/20:0, d16:1/22:0)^#^	Sphingomyelins	−0.91±0.75	0.3±1.19	**2.52×10^−10^**	−0.71±0.7	0.1±1.06	**7.45×10^−07^**	**9.64×10^−05^**	**0.0402**	−0.28±0.96	**7.81×10^−05^**	−0.93±0.91	0.0655
1-Stearoyl-2-arachidonoyl- GPC (18:0/20:4)	Phosphatidylcholine	−0.69±0.62	0.14±1.19	**3.28×10^−06^**	−0.53±0.69	0.26±1.16	**4.62×10^−06^**	**0.002**	**0.0007**	−0.25±0.75	**0.0005**	−0.48±0.85	0.0989
N2,N2-Dimethylguanosine	Purine metabolism, guanine-containing	2±0.69	0.15±0.99	**8.50×10^−21^**	1.9±0.79	0.28±1.18	**5.02×10^−15^**	**4.73×10^−06^**	**1.10×10^−06^**	1.35±0.82	**6.44×10^−07^**	1.81±0.9	0.1246
Sphingomyelin (d18:1/21:0, d17:1/22:0, d16:1/23:0)^#^	Sphingomyelins	−0.87±0.9	0.36±1.18	**8.60×10^−11^**	−0.56±0.74	0.17±1.03	**1.53×10^−06^**	**1.25×10^−05^**	**0.0161**	−0.15±1.32	**0.0032**	−0.77±0.76	0.3203
N-Acetylmethionine	Methionine, cysteine, SAM and taurine metabolism	1.17±0.76	0.18±1.09	**1.42×10^−10^**	1.16±0.75	0.21±1.16	**1.00×10^−08^**	**0.0286**	**0.031**	0.44±1.03	**7.22×10^−08^**	1.12±0.7	0.3691
1-Linoleoyl-2-arachidonoyl- GPC (18:2/20:4n6)^#^	Phosphatidylcholine	−0.78±0.63	0.13±1.2	**1.87×10^−09^**	−0.54±0.55	0.32±1.22	**2.20×10^−10^**	**0.001**	**0.0116**	−0.3±0.66	**9.38×10^−05^**	−0.7±0.92	0.5379
Pseudouridine	Pyrimidine metabolism, uracil-containing	1.63±0.78	0.19±0.97	**8.02×10^−17^**	1.67±0.63	0.26±1.26	**2.29×10^−13^**	**0.0018**	**0.0425**	1.28±0.82	**0.0024**	1.57±1.01	0.8223

Data are presented as mean±sd, unless otherwise stated. The data are scaled to the HC group. Metabolites that are significantly different in CTEPH compared with both HC and DC, independent of confounders and significantly different in CTEPH compared to chronic thromboembolic disease (without pulmonary hypertension) (CTED). Bold type represents statistical significance. IPAH/HPAH: idiopathic and heritable pulmonary arterial hypertension; MTA: methylthioadenosine; GPC: glycerophosphocholine; SAM: *S*-adenosyl methionine. ^#^: probable metabolite identity, but unconfirmed (see methods).

### Metabolite changes associated with PEA surgery in CTEPH patients

We hypothesised that plasma levels of some metabolites relate directly to the consequences of raised pulmonary vascular resistance and associated right ventricle strain; if so, PEA surgery would be expected to correct a subset of altered metabolite levels in CTEPH patients.

37 metabolites distinguished operable CTEPH patients sampled pre-PEA from those sampled post-PEA (supplementary table S5). Additionally, 12 of these metabolites showed a nominally significant change in post-PEA surgery in the paired sample validation analysis (correcting 15–100% of perturbation *versus* healthy controls), with seven meeting multiple test corrections including N2,N2-dimethylguanosine and sphingomyelin-(d18:1/22:1, d18:2/22:0, d16:1/24:1) (supplementary table S5 and figure 4b). Taurine increased in the unpaired samples, but decreased in the paired samples, suggesting that this may be a false positive; the other six metabolites showed consistent directions of change.

### Cardiopulmonary metabolism

**FIGURE 5:**
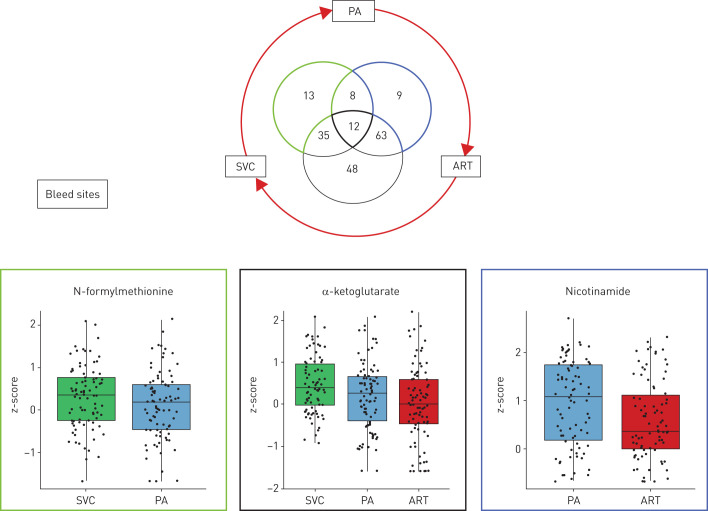
Overlap in metabolites showing significant pulmonary artery (PA)–radial artery (ART), superior vena cava (SVC)–PA and ART–SVC gradients. Boxplots show data for example metabolites from the SVC–PA- (green outline) and PA–ART- (blue outline) specific results and α-ketoglutarate, which was significant across all three gradients.

We hypothesised that cardiac and pulmonary metabolic activity would affect the plasma metabolome and contribute to the metabolic signals observed here in CTEPH and previously in IPAH [5]. We tested this by analysing metabolite gradients across samples from three anatomical arterial and venous sites from patients with IPAH and CTEPH: SVC, PA and ART (figure 5). We found 188 metabolites with significant gradients (p<0.05; supplementary table S6) and the overlap of gradients is depicted in figure 4. Network analysis revealed functionally related clusters of metabolites with tissue gradients that were closely correlated (ρ>0.9) (supplementary figure S2). 21 of the metabolites we have identified as altered in CTEPH also had significant gradients (table 3), including SVC–PA and PA–ART gradients of α-ketoglutarate (tricarboxylic acid (TCA) cycle) and modified methionine metabolites; PA–ART gradients of monohydroxy fatty acids (2-hydroxypalmitate); and SVC–PA gradients of N-formylmethionine and 7-methylguanine (table 3).

**TABLE 3:** Metabolites that associate with chronic thromboembolic pulmonary hypertension (CTEPH) and show significant gradients between sampling sites

	**Subpathway**	**Superpathway**	**Gradients**					
			**PA–ART**		**SVC–PA**		**ART–SVC**	
			**FC**	**FDR p-value**	**FC**	**FDR p-value**	**FC**	**FDR p-value**
**α-Ketoglutarate**	TCA cycle	Energy	−0.184	<0.001	−0.269	<0.001	−0.453	<0.001
**N-Acetylmethionine**	Methionine, cysteine, SAM and taurine metabolism	Amino acid	0.087	0.038	−0.152	<0.001		
**Oxalate (ethanedioate)**	Ascorbate and aldarate metabolism	Cofactors and vitamins	−0.288	<0.001			−0.282	<0.001
**Glycerate**	Glycolysis, gluconeogenesis and pyruvate metabolism	Carbohydrate	−0.36	<0.001			−0.403	<0.001
**2-Hydroxypalmitate**	Fatty acid, monohydroxy	Lipid	−0.304	0.002			−0.414	<0.001
**N-Acetylvaline**	Leucine, isoleucine and valine metabolism	Amino acid	0.118	0.003			0.052	0.026
**N-Formylmethionine**	Methionine, cysteine, SAM and taurine metabolism	Amino acid			−0.185	<0.001		
**7-Methylguanine**	Purine metabolism, guanine containing	Nucleotide			−0.165	<0.001		
**Sphingomyelin (d18:2/23:0, d18:1/23:1, d17:1/24:1)**^#^	Sphingomyelins	Lipid					0.119	<0.001
**γ-Glutamyl-ε-lysine**	γ-Glutamyl amino acid	Peptide					−0.265	0.002
**Sphingomyelin (d18:1/21:0, d17:1/22:0, d16:1/23:0)**^#^	Sphingomyelins	Lipid					0.085	0.005
**N-Acetylserine**	Glycine, serine and threonine metabolism	Amino acid					−0.108	0.008
**1,2-Dilinoleoyl-GPC (18:2/18:2)**	Phosphatidylcholine	Lipid					0.1	0.008
**Androsterone sulfate**	Androgenic steroids	Lipid					0.067	0.012
**1-Stearoyl-2-arachidonoyl-GPC (18:0/20:4)**	Phosphatidylcholine	Lipid					0.145	0.013
**Sphingomyelin (d18:1/22:1, d18:2/22:0, d16:1/24:1)**^#^	Sphingomyelins	Lipid					0.11	0.018
**Tryptophan**	Tryptophan metabolism	Amino acid					0.109	0.018
**N-Acetylalanine**	Alanine and aspartate metabolism	Amino acid					−0.128	0.022
**1-Linoleoyl-2-arachidonoyl-GPC (18:2/20:4n6)**^#^	Phosphatidylcholine	Lipid					0.099	0.03
**N-Acetylphenylalanine**	Phenylalanine metabolism	Amino acid					0.092	0.033
**Sphingomyelin (d18:1/17:0, d17:1/18:0, d19:1/16:0)**	Sphingomyelins	Lipid					0.083	0.045
**1-(1-Enyl-palmitoyl)-2-linoleoyl-GPC (P-16:0/18:2)**^#^	Plasmalogen	Lipid					0.068	0.046

PA: pulmonary artery; ART: radial artery; SVC: superior vena cava; FC: fold change; FDR: false discovery rate (corrected p-values displayed); TCA: tricarboxylic acid; SAM: *S*-adenosyl methionine. ^#^: probable metabolite identity, but unconfirmed (see methods).

In addition, metabolites associated with CTEPH and gradients showed significant associations with clinical measures of disease severity, with the strongest associations observed between metabolites with SVC–PA gradients (*e.g.* N-formylmethionine, N-acetylmethionine and α-ketoglutarate) and measures of adverse clinical outcome (mean right atrial pressure, cardiac output and 6-min walk distance, effect size estimates up to +/-0.432; supplementary figure S3).

We performed an enrichment analysis of the metabolite pathways represented by four or more metabolites (supplementary table S7). TCA cycle metabolites were enriched in both SVC–PA and PA–ART gradients (p<0.05), whereas nicotinamide, nicotinate, phospholipid and lysoplasmalogen metabolites were enriched in PA–ART and ART–SVC gradients (p<0.05). Plasmalogens were enriched only in the PA–ART gradient analysis (p=0.032; supplementary table S7).

### Overlap between metabolites associated with CTEPH, response to surgery and plasma gradients between sampling sites

Overall, we detected several metabolites robustly associated with CTEPH compared to relevant controls and with response to PEA surgery and explored the association of levels of metabolites with passage of blood across different vascular beds. 11 metabolites were overlapping from these main analyses, as summarised in figure 5. In particular, sphingomyelin (d18:1/22:1, d18:2/22:0, d16:1/24:1)* and N2,N2-dimethylguanosine were associated with CTEPH, and changed post-surgery, suggesting a close association with disease development and reversal.

## Discussion

This comprehensive profile of plasma metabolites has identified circulating metabolites that associate with CTEPH and a subset of metabolites that change in response to an effective treatment. The metabolic profile correlates with clinical severity, which together with demonstrating changes in plasma metabolite levels across the lung and heart, provides biological plausibility. Therefore, metabolic profiling may have clinical utility as a noninvasive approach to assessing response to PH treatments.

Most of the metabolic changes seen in CTEPH were notably similar to those observed in IPAH. This included increased modified nucleosides, TCA cycle intermediates, monohydroxy fatty acids, tryptophan, polyamine and arginine metabolites, and decreased sphingomyelin, phosphocholines and steroid metabolites. Differences in metabolite levels between IPAH and CTEPH were subtle and significant for only five metabolites: four modified nucleosides (5-methylthioadenosine, N1-methyladenosine, N1-methylinosine, 7-methylguanine) and N-formylmethionine. Importantly, some of these metabolites (7-methylguanine, N-formylmethionine) also exhibited plasma gradients from the SVC to the PA, which will include metabolites draining from the coronary sinus, indicating a potential relevance to cardiac metabolism, further supported by significant correlations with haemodynamics. RNA modifications are associated with multiple diseases ranging from various types of cancer and immune disease to neurodevelopmental disorders [11–14]. The dynamic and reversible nature of nucleoside modifications identifies these metabolites as candidates to monitor therapeutic response [15], as exemplified by the change in N2,N2-dimethylguanosine following PEA. While many metabolites are affected by age and sex, we found the differences associated with CTEPH were much larger and independent of these and other potential confounders.

The overlap in metabolic disturbance between PAH and CTEPH is understandable, and probably reflects common changes in cardiopulmonary structure and function [16]. Indeed, similarities between CTEPH and PAH with pulmonary arterial remodelling and endothelial cell dysfunction, as well as subsequent right ventricle remodelling are well documented [16, 17]. The implication of this is that future therapeutic strategies which act by correcting the metabolic dysfunction observed could be investigated not just in CTEPH, but potentially in all forms of PH that demonstrate similar metabolic disturbances. When studying CTEPH it is challenging to dissociate the effects of PH and chronic thromboembolism, both of which can affect metabolism. To mitigate this, we included comparisons with patients with chronic thromboembolism without PH, and patients with IPAH. While some effects of the severity and duration of thromboembolism in CTEPH patients may remain, the changes we observe are most likely driven by the haemodynamics of PH and the associated pulmonary vascular remodelling and right heart dysfunction.

We explored the metabolites altered in CTEPH patients sampled post-PEA compared to pre-operative cases and were able to verify correction of six metabolites which report on relevant pathways in patients sampled both prior to and after full recovery from PEA. This included two sphingomyelins which, through structural and signalling roles including cell cholesterol and plasma membrane homeostasis, play an important role in cardiovascular health [18]; here we also show significant inverse correlations with haemodynamic parameters. Reduced α-tocopherol, a potent antioxidant and cytoprotective agent which inhibits platelet aggregation and promotes vasodilation [19, 20] and is reduced in the failing right ventricle [21], was also corrected post-surgery. 3-Ureidopropionate, a pyrimidine breakdown product which can inhibit complex V of the respiratory complex chain [22] was also decreased back towards normal levels post-PEA. The modified nucleoside N2,N2-dimethylguanosine, which could reflect stress or hyperproliferation of vascular cells, was partially corrected by surgery, adding to its utility as a risk marker already established in PAH [5]. The responsiveness of these markers to successful therapy in CTEPH is encouraging for their utility in monitoring successful treatment in other forms of PH.

By sampling PH patients at different anatomical locations, we aimed to characterise alterations in the plasma metabolome across tissues, in particular the heart and lung. In the PA–ART gradients we also saw enrichment in nicotinamide/nicotinate (1-methylnicotinamide has antithrombotic activity [23]), phospholipid, lysoplasmalogen and plasmalogen (antioxidants [24] which can be targeted by hypoxia-induced phospholipases [25]) metabolites, reflecting at least in part, the metabolic activity of the lung. In gradients from the PA–ART and SVC–PA samples we found enrichment of TCA cycle metabolites such as α-ketoglutarate, which was also elevated in PH patients. Previous metabolomic [5, 26] and imaging studies [27] have demonstrated disrupted bioenergetics in IPAH and CTEPH. Accumulation of TCA cycle intermediates is consistent with reduced mitochondrial glucose oxidation, previously reported in PAH and a therapeutic target [28]. Mitochondrial dysfunction in pulmonary artery cells [29], right ventricle [30] and peripheral organs [31] points toward multiorgan energetic reprogramming [32] and is now considered an important component of the pathophysiology of PAH. Our data suggest that this may also be an important feature of CTEPH.

During exercise, fit individuals elevate plasma glycerol (lipolysis), fatty acid entry to the TCA cycle (pantothenate) and expand the TCA cycle intermediate pool [33]. In patients with oxidative phosphorylation dysfunction (mitochondrial/McArdle disease) these responses to increased demand on skeletal muscle are not maintained [34]. Equally, disruption of TCA intermediates and purine metabolites is associated with right ventricular–pulmonary vascular dysfunction in PH [35] and right ventricular fatty acid metabolism is perturbed [36]. We found that metabolites in these and other (modified nucleosides and lysophospholipids) pathways were associated with disease severity and exercise performance in CTEPH patients and further studies such as skeletal muscle biopsy metabolomics may be required to fully appreciate the tissue specificity of these changes. Similarly, differential metabolic response to environmental interventions (diet, exercise programmes) can shed new light on the impact on lifestyle modifications on disease trajectory [37, 38].

While well established in heart failure [39], there is also a growing body of evidence that perturbations in systemic metabolism are involved in the pathogenesis of PAH and CTEPH [40]. This appears to include a role for the gut microbiome in PAH [41] with some bacterial taxa enriched in PAH stool samples and associated microbial metabolite changes in PAH patients [42]. In line with these findings we show here perturbations and significant systemic gradients of microbial metabolites, including those involved in tryptophan, sphingomyelins and phosphatidylcholine metabolism.

The strengths of this study include the large sample size, stringent sampling and processing conditions, inclusion of disease controls, comprehensive clinical assessment, including near normalisation of pulmonary haemodynamics, and its untargeted approach to assessing a wide range of plasma metabolites. There were also limitations. Plasma samples were taken at advanced stages of CTEPH, which makes it difficult to distinguish causative from compensatory changes. The influence of current medical therapies on metabolic profiles was also not assessed. Sampling directly from the coronary sinus could better characterise transcardiac metabolism in future studies. Reduced plasma albumin levels in chronic diseases such as CTEPH more closely represent inflammation, and thus have limited utility in estimating nutritional status. For optimal clinical utility, the effects of diurnal variation and diet, through collection of accurate nutritional data, on specific metabolic profiles will need to be better understood, but some confidence can be taken from pilot data from CTEPH patients sampled in a fasting state who demonstrated several similar perturbations [26].

### Conclusion

We identified a metabolic profile that separates CTEPH from healthy and disease controls, but the overlap in metabolic disturbance between PAH and CTEPH probably reflects common changes in cardiopulmonary structure and function. Plasma metabolite gradients implicate cardiopulmonary tissue metabolism of metabolites associated with PH. Metabolites that respond to surgery with improvement in pulmonary haemodynamics could be a suitable noninvasive marker for evaluating future targeted therapeutic interventions in pulmonary hypertension.

## Supplementary material

10.1183/13993003.03201-2020.Supp1 **Please note:** supplementary material is not edited by the Editorial Office, and is uploaded as it has been supplied by the author.
Supplementary material ERJ-03201-2020.Supplement

## Shareable PDF

10.1183/13993003.03201-2020.Shareable1 This one-page PDF can be shared freely online.
Shareable PDF ERJ-03201-2020.Shareable
