# Supplementary material for: Plasma metabolomics exhibit response to therapy in chronic thromboembolic pulmonary hypertension
Source: Eur Respir J. 2021 Apr 1;57(4):2003201. doi: 10.1183/13993003.03201-2020 (PMC8012591; doi:10.1183/13993003.03201-2020)
Supplement: Supplementary file 1 [file ERJ-03201-2020.Supplement.pdf]

Online data supplement – Supplementary methods, tables and figures

Metabolomics in CTEPH by Swietlik et al.

Table of Contents

Supplementary Methods	2
Supplementary Table S1. Cohort Characteristics for CTEPH analysis.	3-4
Supplementary Table S2. Altered plasma metabolite profiles in CTEPH patients	5-8
Supplementary Table S3. Age and sex effects on CTEPH metabolites	9-11
Supplementary Table S4. ROC analysis	12-13
Supplementary Table S5. Changes in metabolomic profile pre- and post-PEA	14-15
Supplementary Table S6. Full list of metabolites with tissue gradients	16-26
Supplementary Table S7. Enrichment analysis of all pathways.	27-28
Supplementary Figure S1. Age and sex effects on CTEPH metabolites	29-30
Supplementary Figure S2. Relevance network analysis.	31
Supplementary Figure S3. Clinical variables and metabolites	32
Supplementary References	33

Supplementary Methods

Assessment of operability

All patients with chronic thromboembolic pulmonary hypertension (CTEPH) included in the study were assessed in pulmonary endarterectomy (PEA) multidisciplinary team meetings (MDT) involving at least one cardiothoracic surgeon, cardiothoracic radiologist and chest physician. Technical operability was established based on surgical accessibility of thromboembolic material assessed by CT pulmonary angiogram and ventilation perfusion scan, in conjunction with thrombus burden and PVR.

PEA surgical procedure

PEA involves removal of obstructive thromboembolic material from the pulmonary arteries including the intima and superficial media in order to reduce PVR, decrease right ventricle (RV) afterload and improve ventilation-perfusion mismatch. PEA was performed through median sternotomy with cardiopulmonary bypass (CPB) enabling hypothermia to 20℃ and safe circulatory arrest(1). Deep hypothermic circulatory arrest (DHCA) provided a clear operating field and was limited to 20 minutes intervals on each side. The identification of endarterectomy plane was followed by distal dissection to remove endarterectomy specimen as previously described(2, 3).

Right heart catheterisation (RHC) procedure and sampling at 3 anatomical locations

All patients sampled at 3 anatomical locations had elective RHC performed via right internal jugular (RIJ) vein. The access area was cleaned with 2% chlorhexidine and infiltrated with 6 to 9 ml of local aesthetic (1% lignocaine), position of RIJ was confirmed with ultrasound under sterile conditions. 7 French sheath was introduced to the RIJ using a Seldinger technique. Pressure transducer was set to zero at mid-thoracic level(1). Swan Ganz catheter was inserted and the position of the catheter was confirmed by pressure trace and fluoroscopy screening. First sample was obtained from SVC with the catheter tip just above right atrium entry, subsequently catheter was advanced and RA pressure was measured. Next the catheter was floated to the RV, where RV systolic pressure (RVSP) and end-diastolic pressure were recorded. Upon advancing the catheter to pulmonary artery (PA) systolic and diastolic pressure were measured (sPAP and dPAP) and the PA blood sample was obtained for the metabolomic analysis. After that the balloon was inflated and advanced to the distal portion of pulmonary artery in order to obtain pulmonary artery wedge pressure (PAWP). Subsequently cardiac output was measured using thermodilution technique. Saturation run was performed at the end of RHC while removing the catheter, 3 ml samples for oximetry analysis were taken from PA, RV, RA and SVC. Finally, peripheral arterial sample for metabolomics analysis was obtained from distal portion of radial artery.

	Operable CTEPH sampled pre-PEA	Operable CTEPH sampled post-PEA	Operable CTEPH with paired samples pre/post-PEA	p-value
	N=64	N=82	N=43	
Baseline characteristics				
Age at sampling [years]	65 [50;74]	67 [54;75]	63 [55;72]	0.416
Time from sampling to PEA [months]	8.7[12.7;6.9]		7.7[5.6;9.9]	
Sex: F	23 (36%)	37 (45%)	15 (35%)	0.406
Ethnicity: European	41 (64%)	69 (84%)	37 (86%)	0.005
BMI [kg/m^2]	28 [25;31]	29 [25;33]	28 [25;33]	0.617
WHO functional class:				0.022
I	4 (7%)	12 (15%)	1 (2%)	
II	15 (25%)	29 (36%)	8 (19%)	
III	39 (65%)	35 (44%)	31 (72%)	
IV	2 (3%)	4 (5%)	3 (7%)	
6MWD [m]	240 [96;384]	382 [286;427]	336 [240;363]	0.002
Creatinine [mmol/l]	90 [73;103]	79 [70;90]	86 [75;100]	0.063
Bilirubin [μmol/l]	12 [9;19]	10 [8;14]	15 [9;20]	0.056
mRAP[mmHg]	8 [6;13]	10 [7;14]	8 [6;13]	0.076
mPAP[mmHg]	44 (14)	48 (11)	43 (12)	0.075
mPAWP[mmHg]	12 [9;16]	13 [10;15]	10 [9;12]	0.064
PVR[WU]	7.1 [4.5;10.6]	7.8 [5.2;11.5]	7.9 [5.9;10.3]	0.807
CO[L/min]	4.1 [3.4;5.0]	3.8 [2.9;4.8]	4.0 [3.5;4.5]	0.494
COPD	7 (11%)	12 (15%)	3 (7%)	0.437
Diabetes	5 (8%)	5 (6%)	6 (14%)	0.330
Atherosclerosis	17 (27%)	20 (24%)	12 (55%)	0.019
Atrial arrhythmia	11 (17%)	23 (28%)	8 (19%)	0.238
Hypertension	19 (30%)	26 (32%)	9 (21%)	0.435
Dyslipidemia	17 (27%)	12 (15%)	11 (26%)	0.156
Post pulmonary endarterectomy				
Time from PEA to sampling [months]		37[11.8;65.2]	5.8[4.7;11.9]	
mPAP [mmHg]		28 (11)	28 (10)	
PVR [WU]		1[0.99;3.9]	1.8 [0.97;3.4]	

Supplementary Table S1. Cohort Characteristics for CTEPH analysis. Significance is shown using Kruskal-Wallis (continuous) and Chi-squared tests (categorical) showing only minor differences in baseline characteristics between groups. Means and standard deviations, median and IQR and counts are given. BMI, body mass index; WHO, World Health Organisation; 6MWD, six-minute walk distance; mRAP, mean right atrial pressure; mPAP, mean pulmonary artery pressure; mPAWP, mean pulmonary artery wedge pressure; PVR, pulmonary artery resistance; CO, cardiac output; COPD, chronic obstructive

pulmonary disease; PEA, pulmonary endarterectomy. Ethnicity is shown for subjects who self-declared.

		Discovery			Validation			Linear regression with confounders			Comparator groups			
**Metabolite**	**Metabolic pathway**	**CTEPH**	**HC**	**Sig.**	**CTEPH**	**HC**	**Sig.**	**HC vs CTEPH**	**Main confounder vs HC**	**DC vs CTEPH**	**CTED**	**Sig.**	**IPAH**	**Sig.**
		mean (SD)	mean (SD)		mean (SD)	mean (SD)		**Sig.**		**Sig.**	mean (SD)		mean (SD)	
**Significant in all analyses**														
5-methylthioadenosine (MTA)	Polyamine Metabolism	1.72 (0.84)	0.07 (1.22)	**5.00E-17**	1.55 (0.91)	0.32 (1.12)	**6.95E-12**	**5.21E-05**		**0.0009**	1.34 (0.73)	**0.0019**	0.86 (1.26)	**3.75E-13**
N1-methyladenosine	Purine Metabolism, Adenine containing	1.58 (0.78)	0.03 (1.01)	**3.02E-17**	1.67 (0.61)	0.4 (1.1)	**8.10E-14**	**4.55E-05**		**0.0105**	0.91 (1.08)	**2.07E-06**	1.21 (0.93)	**1.56E-06**
N1-methylinosine	Purine Metabolism, (Hypo)Xanthine/Inosine containing	1.64 (1.54)	0 (1.1)	**1.51E-12**	1.91 (1.45)	0.41 (1.06)	**8.70E-13**	**7.20E-05**		**2.35E-05**	1.59 (1.11)	**0.0406**	1.65 (1.11)	**0.008**
7-methylguanine	Purine Metabolism, Guanine containing	1.23 (1.09)	0.01 (1.16)	**8.47E-10**	1.27 (1.25)	0.45 (1.19)	**0.0001**	**0.0007**		**0.0099**	0.54 (1.08)	**4.18E-05**	0.95 (1.28)	**0.019**
N-formylmethionine	Methionine, Cysteine, SAM and Taurine Metabolism	1.45 (0.88)	0.05 (1.02)	**6.10E-14**	1.51 (0.78)	0.36 (1.11)	**4.27E-11**	**0.0024**		**0.0042**	1.22 (0.78)	**0.014**	1.31 (0.86)	**0.0406**
**Significant versus HC, DC and CTED**														
sphingomyelin (d18:1/20:0, d16:1/22:0)*	Sphingomyelins	-0.91 (0.75)	0.3 (1.19)	**2.52E-10**	-0.71 (0.7)	0.1 (1.06)	**7.45E-07**	**9.64E-05**		**0.0402**	-0.28 (0.96)	**7.81E-05**	-0.93 (0.91)	0.0655
1-stearoyl-2-arachidonoyl-GPC (18:0/20:4)	Phosphatidylcholine (PC)	-0.69 (0.62)	0.14 (1.19)	**3.28E-06**	-0.53 (0.69)	0.26 (1.16)	**4.62E-06**	**0.002**		**0.0007**	-0.25 (0.75)	**0.0005**	-0.48 (0.85)	0.0989
N2,N2-dimethylguanosine	Purine Metabolism, Guanine containing	2 (0.69)	0.15 (0.99)	**8.50E-21**	1.9 (0.79)	0.28 (1.18)	**5.02E-15**	**4.73E-06**		**1.10E-06**	1.35 (0.82)	**6.44E-07**	1.81 (0.9)	0.1246
sphingomyelin (d18:1/21:0, d17:1/22:0, d16:1/23:0)*	Sphingomyelins	-0.87 (0.9)	0.36 (1.18)	**8.60E-11**	-0.56 (0.74)	0.17 (1.03)	**1.53E-06**	**1.25E-05**		**0.0161**	-0.15 (1.32)	**0.0032**	-0.77 (0.76)	0.3203
N-acetylmethionine	Methionine, Cysteine, SAM and Taurine Metabolism	1.17 (0.76)	0.18 (1.09)	**1.42E-10**	1.16 (0.75)	0.21 (1.16)	**1.00E-08**	**0.0286**		**0.031**	0.44 (1.03)	**7.22E-08**	1.12 (0.7)	0.3691
1-linoleoyl-2-arachidonoyl-GPC (18:2/20:4n6)*	Phosphatidylcholine (PC)	-0.78 (0.63)	0.13 (1.2)	**1.87E-09**	-0.54 (0.55)	0.32 (1.22)	**2.20E-10**	**0.001**		**0.0116**	-0.3 (0.66)	**9.38E-05**	-0.7 (0.92)	0.5379
pseudouridine	Pyrimidine Metabolism, Uracil containing	1.63 (0.78)	0.19 (0.97)	**8.02E-17**	1.67 (0.63)	0.26 (1.26)	**2.29E-13**	**0.0018**		**0.0425**	1.28 (0.82)	**0.0024**	1.57 (1.01)	0.8223

Significant versus HC, DC independent of confounders														
1,2-dilinoleoyl-GPC (18:2/18:2)	Phosphatidylcholine (PC)	-0.78 (0.75)	0.11 (1.25)	**5.91E-07**	-0.7 (0.67)	0.36 (1)	**7.68E-11**	**0.0025**		**0.0069**	-0.54 (0.79)	0.1446	-0.58 (0.93)	0.1023
N-acetylphenylalanine	Phenylalanine Metabolism	1 (0.94)	0.11 (1.11)	**9.94E-08**	0.98 (1.05)	0.27 (1.13)	**5.42E-05**	**0.0217**		**0.0029**	1.11 (0.88)	0.3722	0.85 (0.94)	0.1535
gamma-glutamyl-epsilon-lysine	Gamma-glutamyl Amino Acid	-1.06 (1.03)	0.33 (1.35)	**2.38E-09**	-0.98 (1.05)	0.05 (1.43)	**7.68E-06**	**0.0169**		**0.0464**	-1.11 (0.91)	0.4414	-0.35 (1.15)	1.82E-09
sphingomyelin (d18:1/17:0, d17:1/18:0, d19:1/16:0)	Sphingomyelins	-0.7 (1)	0.35 (1.15)	**4.63E-08**	-0.54 (0.85)	0.14 (1.21)	**0.0001**	**0.0239**		**0.0273**	-0.66 (1.19)	0.5115	-0.5 (1.05)	0.4819
oxalate (ethanedioate)	Ascorbate and Aldarate Metabolism	-0.7 (1.07)	0.28 (1)	**4.13E-08**	-0.92 (0.91)	0.18 (1.29)	**8.36E-08**	**0.0087**		**0.0023**	-0.8 (1.25)	0.5194	-0.54 (1.04)	0.009
1-(1-enyl-palmitoyl)-2-linoleoyl-GPC (P-16:0/18:2)*	Plasmalogen	-0.95 (1.06)	0.27 (1.17)	**3.17E-09**	-1.08 (1.24)	0.16 (1.21)	**6.04E-08**	**0.0065**		**0.0446**	-0.87 (1.33)	0.6299	-0.99 (1.12)	0.5948
2-hydroxypalmitate	Fatty Acid, Monohydroxy	1.08 (0.86)	0.29 (1.12)	**8.56E-06**	0.94 (0.99)	0.2 (1.19)	**5.34E-05**	**0.0002**		**0.0005**	1.01 (0.91)	0.909	0.85 (0.95)	0.052
**Significant versus HC independent of confounders**														
sphingomyelin (d18:1/22:1, d18:2/22:0, d16:1/24:1)*	Sphingomyelins	-0.87 (0.81)	0.34 (1.24)	**7.45E-10**	-0.71 (0.82)	0.1 (1)	**5.21E-07**	**0.0002**		0.0549	0 (0.86)	8.20E-09	-0.98 (0.97)	0.018
dimethylarginine (SDMA + ADMA)	Urea cycle; Arginine and Proline Metabolism	1.3 (1.02)	0.08 (1.09)	**1.04E-10**	1.24 (0.89)	0.34 (1.04)	**4.49E-08**	**0.0003**		0.0596	0.86 (1.07)	0.0043	1.07 (1.02)	0.0264
methionine sulfone	Methionine, Cysteine, SAM and Taurine Metabolism	1.22 (1.39)	0.22 (1.1)	**1.30E-05**	1.58 (1.3)	0.17 (1.14)	**6.98E-10**	**0.0025**		0.0606	0.28 (1.26)	3.34E-07	1.75 (1.27)	0.006
kynurenine	Tryptophan Metabolism	1.24 (0.95)	0.12 (1.13)	**1.62E-08**	1.37 (1.12)	0.31 (1.31)	**1.42E-06**	**0.0159**		0.0694	0.88 (1.38)	0.1004	1.25 (1.11)	0.6286
androsterone sulfate	Androgenic Steroids	-1.28 (1.28)	0.26 (1.19)	**2.59E-11**	-1.43 (1.43)	0.2 (1.47)	**4.26E-10**	**0.0005**		0.0728	-0.45 (1.5)	2.57E-05	-1.28 (1.36)	0.5555
sphingomyelin (d18:2/23:0, d18:1/23:1, d17:1/24:1)*	Sphingomyelins	-0.61 (0.81)	0.39 (1.05)	**1.90E-10**	-0.33 (0.68)	0.18 (0.9)	**9.03E-06**	**0.0002**		0.075	-0.33 (1.12)	0.286	-0.48 (0.66)	0.9952
histidine	Histidine Metabolism	-1.27 (1.33)	0.19 (1.03)	**7.41E-11**	-1.53 (1.5)	0.26 (1.32)	**6.33E-11**	**0.039**		0.0869	-0.14 (1.34)	1.85E-08	-1.53 (1.33)	0.4164
alpha-ketoglutarate	TCA Cycle	1.06 (1.27)	0.25 (1.18)	**3.44E-07**	1.05 (1.31)	0.25 (1.44)	**0.0001**	**0.0274**		0.1066	1.02 (0.7)	0.0712	1.13 (1.18)	0.7675
oleoyl ethanolamide	Endocannabinoid	1.57 (0.85)	0.38 (1.19)	**5.06E-10**	1.17 (1.13)	0.04 (1.13)	**3.47E-09**	**0.0001**		0.177	1.45 (1.08)	0.6262	0.95 (0.97)	8.56E-08
glycerate	Glycolysis, Gluconeogenesis, and Pyruvate Metabolism	-0.72 (0.99)	0.48 (1.15)	**1.88E-10**	-0.91 (0.95)	-0.06 (1.26)	**2.38E-06**	**0.029**		0.2477	-1 (1.25)	0.0857	-0.57 (1.05)	0.0087

C-glycosyltryptophan	Tryptophan Metabolism	1.2 (0.75)	0.17 (0.92)	**3.47E-11**	1.31 (0.81)	0.24 (1.15)	**3.97E-09**	**0.0062**		0.3142	0.24 (0.97)	6.11E-12	1.27 (0.94)	0.5221
N-acetylserine	Glycine, Serine and Threonine Metabolism	1.49 (1.04)	0.09 (0.93)	**2.19E-12**	1.54 (1.08)	0.37 (1.18)	**3.28E-08**	**0.0271**		0.3199	0.76 (1.05)	5.36E-06	1.41 (1.17)	0.5923
N-acetylvaline	Leucine, Isoleucine and Valine Metabolism	0.96 (0.82)	0.1 (1.11)	**1.33E-08**	1.01 (0.69)	0.26 (0.98)	**1.24E-07**	**0.0397**		0.3923	0.86 (0.75)	0.2182	0.91 (0.89)	0.5789
behenoyl sphingomyelin (d18:1/22:0)*	Sphingomyelins	-0.68 (0.67)	0.34 (0.98)	**2.37E-11**	-0.47 (0.63)	0.19 (1.06)	**4.16E-06**	**0.0049**		0.4009	-0.1 (0.67)	1.62E-06	-0.65 (0.78)	0.0769
N-acetylalanine	Alanine and Aspartate Metabolism	1.1 (0.95)	0.15 (0.94)	**1.52E-10**	1.18 (0.79)	0.28 (1.17)	**5.16E-07**	**0.0423**		0.5963	0.8 (0.89)	0.0025	1 (0.95)	0.249
tryptophan	Tryptophan Metabolism	-0.44 (0.87)	0.22 (1.12)	**9.54E-05**	-0.48 (0.91)	0.22 (1.31)	**0.0001**	**0.0405**		0.6793	0.05 (0.98)	0.0003	-0.28 (1.07)	0.0634
**Significant versus HC but dependent on confounders**														
1-linoleoyl-GPC (18:2)	Lysophospholipid	-0.92 (0.68)	0.16 (1.2)	**1.95E-09**	-0.86 (0.68)	0.28 (1.2)	**1.09E-09**	0.0662	Age	0.7221	-0.7 (1.04)	0.1358	-1.07 (1.05)	0.0082
citrate	TCA Cycle	1.58 (1.13)	-0.01 (1.15)	**2.63E-12**	1.33 (1.15)	0.41 (1.12)	**6.09E-06**	0.0775	PDE5 inhibitors	0.0486	1.1 (1.18)	0.038	0.93 (1.24)	5.14E-06
1-palmitoyl-GPC (16:0)	Lysophospholipid	-0.84 (0.64)	0.24 (1.08)	**8.40E-11**	-0.9 (0.74)	0.21 (1.29)	**1.37E-09**	0.0972	Bilirubin	0.0031	-0.13 (0.78)	5.49E-10	-0.69 (0.91)	0.0722
malate	TCA Cycle	1.34 (0.8)	0 (1.37)	**3.98E-11**	1.31 (0.88)	0.35 (0.83)	**6.46E-10**	0.1074	Age	0.013	0.53 (0.81)	3.46E-09	1.32 (0.93)	0.9231
4-acetamidobutanoate	Polyamine Metabolism	1.57 (1.2)	0.14 (0.98)	**7.11E-13**	1.58 (1.13)	0.28 (1.22)	**2.32E-09**	0.1192	Age	0.6594	0.35 (1.73)	5.58E-07	1.63 (1.22)	0.4758
erythronate*	Aminosugar Metabolism	0.89 (0.79)	0.11 (0.94)	**3.97E-07**	1.07 (0.77)	0.25 (1.21)	**2.47E-06**	0.1233	Diuretics	0.7631	0.58 (1.04)	0.0077	0.94 (0.99)	0.7118
leucine	Leucine, Isoleucine and Valine Metabolism	-0.59 (1.09)	0.15 (1.02)	**0.0001**	-0.54 (1.02)	0.27 (1.23)	**3.20E-05**	0.1256	Gender	0.3889	0.35 (0.99)	7.31E-08	-0.59 (1.01)	0.9785
1-stearoyl-2-linoleoyl-GPC (18:0/18:2)*	Phosphatidylcholine (PC)	-0.78 (0.9)	0.11 (1.1)	**7.41E-07**	-0.64 (0.87)	0.32 (1.17)	**4.07E-07**	0.1405	Statins	0.0817	-0.33 (0.63)	0.0006	-0.59 (1.05)	0.2836
arginine	Urea cycle; Arginine and Proline Metabolism	-1.16 (1.05)	0.22 (1.2)	**2.79E-11**	-1.11 (0.82)	0.26 (1.19)	**1.02E-12**	0.1406	Age	0.0507	-0.95 (1.38)	0.0765	-1.1 (1.15)	0.9618
1-stearoyl-GPC (18:0)	Lysophospholipid	-0.9 (0.63)	0.21 (1.18)	**2.05E-09**	-0.84 (0.73)	0.24 (1.24)	**4.38E-09**	0.1595	Bilirubin	0.1739	-0.18 (0.92)	1.25E-07	-0.73 (0.84)	0.2863
homoarginine	Urea cycle; Arginine and Proline Metabolism	-0.8 (1.11)	0.24 (1.13)	**8.63E-08**	-1.18 (1.04)	0.23 (1.46)	**9.63E-10**	0.1746	Age	0.8135	0.1 (1.1)	5.25E-09	-0.99 (1.14)	0.8129

N-acetylneuraminate	Aminosugar Metabolism	0.87 (0.71)	0.08 (1.14)	**5.10E-06**	0.87 (0.85)	0.28 (1.03)	**6.04E-05**	0.181	Age	0.0414	-0.08 (0.72)	2.74E-13	0.91 (0.77)	0.7097
orotidine	Pyrimidine Metabolism, Orotate containing	0.92 (0.72)	0.1 (1.12)	**1.14E-07**	1.03 (0.66)	0.27 (1.21)	**2.56E-06**	0.2776	Gender	0.19	0.45 (0.98)	1.10E-05	1.1 (0.82)	0.0021
N6-carbamoylthreonyladenosine	Purine Metabolism, Adenine containing	1.35 (1.01)	0.14 (1.06)	**1.54E-12**	1.43 (0.89)	0.32 (1.3)	**1.18E-09**	0.2958	Age	0.1371	1.11 (0.84)	0.0028	1.32 (0.89)	0.2009
1-(1-enyl-palmitoyl)-GPC (P-16:0)*	Lysoplasmalogen	-0.51 (0.9)	0.28 (1.29)	**4.64E-05**	-0.63 (1)	0.18 (1.06)	**2.36E-06**	0.4844	Antidiabetic	0.9232	0.02 (0.96)	9.18E-05	-0.57 (0.89)	0.6294
methionine sulfoxide	Methionine, Cysteine, SAM and Taurine Metabolism	-0.61 (0.84)	0.12 (1.08)	**4.23E-06**	-0.61 (0.98)	0.23 (1.27)	**2.27E-05**	0.4864	Gender	0.3433	-0.68 (0.77)	0.6097	-0.11 (0.87)	2.02E-09
asparagine	Alanine and Aspartate Metabolism	-0.6 (0.95)	0.19 (1.12)	**1.02E-05**	-0.87 (0.83)	0.2 (1.4)	**2.87E-07**	0.5035	Age	0.0657	-0.59 (1.05)	0.549	-0.87 (1.02)	0.1567
1-arachidonoyl-GPC (20:4n6)*	Lysophospholipid	-0.6 (1.04)	0.12 (1.4)	**3.57E-05**	-0.54 (1.13)	0.25 (1.11)	**4.12E-05**	0.5114	Statins	0.9221	0.26 (1.04)	2.27E-06	-0.85 (1.07)	0.0125
3-hydroxy-3-methylglutarate	Mevalonate Metabolism	0.92 (0.73)	0.11 (1.11)	**3.50E-07**	0.84 (0.9)	0.21 (1.13)	**0.0001**	0.653	Age	0.5855	0.19 (0.84)	9.72E-09	0.93 (0.81)	0.5036
vanillylmandelate (VMA)	Tyrosine Metabolism	1.53 (1.21)	0.01 (1.13)	**9.88E-12**	1.34 (1.27)	0.4 (1.11)	**5.16E-07**	0.8066	Age	0.1236	0.54 (1.08)	5.68E-07	1.31 (1.3)	0.3765

Supplementary Table S2. Altered plasma metabolite profiles in CTEPH patients.

Metabolites distinguishing CTEPH from healthy and disease controls. 55 metabolites that are significantly different between CTEPH and healthy controls in a discovery and validation cohort ($p<1.54x10^{-4}$) are shown. Mean values are given and the data is scaled to the healthy control group. Significance from linear regression is shown (p value), and for metabolites with p>0.05 in CTEPH HC linear regression, the significant confounder is shown. Significance is also shown for Mann Whitney U test between all CTEPH patients versus CTED and PE patients. *probable metabolite identity, but unconfirmed (see methods). DM, diabetes; GPC, glycerophosphocholine; HC, healthy controls; DC, disease controls; BMI, body mass index; CTED, chronic thromboembolic disease; PE, pulmonary embolism.

Metabolite	Metabolite family	Metabolic pathway	Effect of Age	SE	Sig	Effect of male sex	SE	Sig.	Effect of CTEPH vs HC	SE	Sig.
N1-methylinosine	Nucleotide	Purine Metabolism, (Hypo)Xanthine/Inosine containing	0.02	0.01	0.013	0.31	0.18	0.086	2.03	0.50	7E-05
5-methylthioadenosine (MTA)	Amino Acid	Polyamine Metabolism	0.00	0.01	0.561	0.33	0.15	0.029	1.72	0.42	5E-05
N1-methyladenosine	Nucleotide	Purine Metabolism, Adenine containing	0.01	0.00	0.05	-0.08	0.13	0.55	1.48	0.35	5E-05
7-methylguanine	Nucleotide	Purine Metabolism, Guanine containing	0.01	0.01	0.046	0.24	0.15	0.114	1.46	0.43	0.0007
N-formylmethionine	Amino Acid	Methionine, Cysteine, SAM and Taurine Metabolism	0.01	0.00	0.002	0.11	0.13	0.409	1.11	0.36	0.0024
N2,N2-dimethylguanosine	Nucleotide	Purine Metabolism, Guanine containing	0.02	0.00	1E-04	-0.07	0.12	0.55	1.55	0.33	5E-06
pseudouridine	Nucleotide	Pyrimidine Metabolism, Uracil containing	0.01	0.00	6E-04	0.16	0.13	0.214	1.10	0.35	0.0018
N-acetylmethionine	Amino Acid	Methionine, Cysteine, SAM and Taurine Metabolism	0.00	0.00	0.375	-0.19	0.14	0.164	0.84	0.38	0.0286
1-stearoyl-2-arachidonoyl-GPC (18:0/20:4)	Lipid	Phosphatidylcholine (PC)	-0.01	0.00	0.056	-0.08	0.12	0.528	-1.05	0.33	0.002
1-linoleoyl-2-arachidonoyl-GPC (18:2/20:4n6)*	Lipid	Phosphatidylcholine (PC)	-0.02	0.00	7E-07	0.04	0.13	0.768	-1.17	0.35	0.001
sphingomyelin (d18:1/20:0, d16:1/22:0)*	Lipid	Sphingomyelins	0.00	0.00	0.572	-0.39	0.11	8E-04	-1.26	0.32	1E-04

sphingomyelin (d18:1/21:0, d17:1/22:0, d16:1/23:0)*	Lipid	Sphingomyelins	0.00	0.00	0.647	-0.57	0.12	6E-06	-1.53	0.34	1E-05
2-hydroxypalmitate	Lipid	Fatty Acid, Monohydroxy	0.01	0.00	0.229	-0.02	0.15	0.889	1.52	0.41	0.0002
N-acetylphenylalanine	Amino Acid	Phenylalanine Metabolism	0.00	0.01	0.898	0.25	0.16	0.126	1.03	0.45	0.0217
sphingomyelin (d18:1/17:0, d17:1/18:0, d19:1/16:0)	Lipid	Sphingomyelins	0.01	0.00	0.304	-0.59	0.15	7E-05	-0.92	0.41	0.0239
1,2-dilinoleoyl-GPC (18:2/18:2)	Lipid	Phosphatidylcholine (PC)	-0.01	0.00	7E-04	-0.12	0.13	0.34	-1.09	0.36	0.0025
gamma-glutamyl-epsilon-lysine	Peptide	Gamma-glutamyl Amino Acid	-0.01	0.01	0.225	0.03	0.17	0.864	-1.14	0.47	0.0169
oxalate (ethanedioate)	Cofactors and Vitamins	Ascorbate and Aldarate Metabolism	0.00	0.01	0.436	-0.32	0.16	0.044	-1.18	0.44	0.0087
1-(1-enyl-palmitoyl)-2-linoleoyl-GPC (P-16:0/18:2)*	Lipid	Plasmalogen	-0.01	0.01	0.024	-0.55	0.16	5E-04	-1.19	0.43	0.0065
oleoyl ethanolamide	Lipid	Endocannabinoid	0.00	0.00	0.395	-0.25	0.14	0.071	1.50	0.38	0.0001
dimethylarginine (SDMA + ADMA)	Amino Acid	Urea cycle; Arginine and Proline Metabolism	0.02	0.00	0.001	0.03	0.14	0.813	1.45	0.39	0.0003
methionine sulfone	Amino Acid	Methionine, Cysteine, SAM and Taurine Metabolism	0.01	0.01	0.28	-0.48	0.17	0.004	1.41	0.46	0.0025
alpha-ketoglutarate	Energy	TCA Cycle	0.00	0.01	0.77	-0.20	0.19	0.3	1.19	0.54	0.0274
kynurenine	Amino Acid	Tryptophan Metabolism	0.01	0.01	0.279	0.26	0.16	0.098	1.06	0.44	0.0159
C-glycosyltryptophan	Amino Acid	Tryptophan Metabolism	0.02	0.00	3E-05	-0.17	0.12	0.169	0.94	0.34	0.0062
N-acetylserine	Amino Acid	Glycine, Serine and Threonine Metabolism	0.02	0.01	1E-04	0.11	0.15	0.473	0.93	0.42	0.0271
N-acetylalanine	Amino Acid	Alanine and Aspartate Metabolism	0.01	0.00	0.005	0.03	0.13	0.808	0.77	0.37	0.0423

N-acetylvaline	Amino Acid	Leucine, Isoleucine and Valine Metabolism	0.01	0.00	0.069	0.07	0.13	0.594	0.74	0.36	0.0397
tryptophan	Amino Acid	Tryptophan Metabolism	-0.02	0.00	6E-07	0.18	0.13	0.188	-0.76	0.37	0.0405
behenoyl sphingomyelin (d18:1/22:0)*	Lipid	Sphingomyelins	-0.01	0.00	0.013	-0.30	0.11	0.006	-0.86	0.30	0.0049
histidine	Amino Acid	Histidine Metabolism	-0.03	0.01	1E-06	0.05	0.17	0.769	-1.00	0.48	0.039
sphingomyelin (d18:2/23:0, d18:1/23:1, d17:1/24:1)*	Lipid	Sphingomyelins	0.00	0.00	0.414	-0.81	0.10	5E-15	-1.00	0.27	0.0002
glycerate	Carbohydrate	Glycolysis, Gluconeogenesis, and Pyruvate Metabolism	0.00	0.01	0.871	-0.09	0.17	0.57	-1.02	0.46	0.029
sphingomyelin (d18:1/22:1, d18:2/22:0, d16:1/24:1)*	Lipid	Sphingomyelins	0.00	0.00	0.636	-0.81	0.11	2E-11	-1.19	0.31	0.0002
androsterone sulfate	Lipid	Androgenic Steroids	-0.02	0.01	0.003	0.84	0.17	3E-06	-1.71	0.48	0.0005

Supplementary Table S3 – Effect of age and sex on metabolites associated with CTEPH Linear regression analyses were used to model age and sex effects in healthy controls and CTEPH patients correcting for CTEPH status and confounders.

CTEPH (n=200) vs HC (n=121)	Area Under the Curve	95% Confidence Interval					
Test Result Variable(s)		Lower	Upper	Sig.	Best cut-off	Sensitivity	Specificity
7-methylguanine	0.812	0.766	0.858	7.94E-21	0.83	0.68	0.818
N-formylmethionine	0.878	0.84	0.916	8.1E-30	0.94	0.815	0.843
N1-methyladenosine	0.909	0.875	0.943	1.12E-34	1.14	0.785	0.901
N1-methylinosine	0.871	0.831	0.911	7.68E-29	1.29	0.745	0.95
5-methylthioadenosine (MTA)	0.904	0.87	0.937	7.99E-34	0.89	0.87	0.868
1-stearoyl-2-arachidonoyl-GPC (18:0/20:4)	0.356	0.291	0.421	1.5E-05	0.44	0.92	0.347
1-linoleoyl-2-arachidonoyl-GPC (18:2/20:4n6)*	0.291	0.229	0.354	3.8E-10	0.08	0.84	0.521
sphingomyelin (d18:1/21:0, d17:1/22:0, d16:1/23:0)*	0.31	0.251	0.369	1.17E-08	-0.51	0.565	0.752
sphingomyelin (d18:1/20:0, d16:1/22:0)*	0.308	0.248	0.368	7.73E-09	-0.43	0.63	0.669
N-acetylmethionine	0.864	0.825	0.904	7.01E-28	0.83	0.79	0.851
pseudouridine	0.92	0.89	0.95	1.6E-36	0.83	0.885	0.826
N2,N2-dimethylguanosine	0.936	0.908	0.963	4.03E-39	1.35	0.835	0.942

CTEPH (n=200) vs DC (n=132)	Area Under the Curve	95% Confidence Interval					
Test Result Variable(s)		Lower	Upper	Sig.	Best cut-off	Sensitivity	Specificity
7-methylguanine	0.718	0.663	0.774	1.63E-11	0.74	0.705	0.682
N-formylmethionine	0.746	0.689	0.803	2.96E-14	0.94	0.815	0.636
N1-methyladenosine	0.76	0.706	0.815	1E-15	1.14	0.785	0.659
N1-methylinosine	0.731	0.674	0.787	1.14E-12	1.65	0.69	0.735
5-methylthioadenosine (MTA)	0.732	0.675	0.79	7.63E-13	1.16	0.81	0.614
1-stearoyl-2-arachidonoyl-GPC (18:0/20:4)	0.349	0.286	0.411	3.1E-06	-0.13	0.72	0.553
1-linoleoyl-2-arachidonoyl-GPC (18:2/20:4n6)*	0.384	0.319	0.449	0.000333	-0.46	0.585	0.652
sphingomyelin (d18:1/21:0, d17:1/22:0, d16:1/23:0)*	0.417	0.353	0.481	0.010438	-0.05	0.74	0.439
sphingomyelin (d18:1/20:0, d16:1/22:0)*	0.416	0.351	0.481	0.009658	-0.21	0.695	0.5
N-acetylmethionine	0.739	0.682	0.795	1.74E-13	0.83	0.79	0.636
pseudouridine	0.73	0.67	0.789	1.45E-12	0.97	0.86	0.568
N2,N2-dimethylguanosine	0.769	0.713	0.825	1.16E-16	1.35	0.835	0.682

Supplementary Table S4 – ROC analysis and best cut-offs for metabolites which distinguish CTEPH from healthy and disease controls and CTED patients. Best cut-offs derived using the Youden Index of specificity+sensitivity. Areas under the curve below 0.5 indicate that lower levels of the metabolite are associated with CTEPH – comparable AUCs can be calculated by subtracting these from 1.

Metabolite	Super metabolic pathway	Sub metabolic pathway	CTEPH operable	CTEPH-PostPEA	Sig.	Paired CTEPH, pre-PEA	Paired CTEPH, post-PEA	Sig.	q FDR
sphingomyelin (d18:1/22:1, d18:2/22:0, d16:1/24:1)*	Lipid	Sphingomyelins	-0.85 (0.88)	-0.59 (0.87)	0.043	-0.84	-0.38	0.0002	**0.0075**
taurine	Amino Acid	Methionine, Cysteine, SAM and Taurine Metabolism	-0.35 (0.9)	-0.01 (1.12)	0.0407	0.43	-0.22	0.0007	**0.0131**
sphingomyelin (d18:1/14:0, d16:1/16:0)*	Lipid	Sphingomyelins	-0.45 (0.63)	-0.16 (0.67)	0.0064	-0.61	-0.31	0.0014	**0.0133**
3-ureidopropionate	Nucleotide	Pyrimidine Metabolism, Uracil containing	0.22 (1.56)	-0.03 (1.15)	0.038	1.20	0.74	0.0014	**0.0133**
alpha-tocopherol	Cofactors and Vitamins	Tocopherol Metabolism	0.29 (1.02)	0.62 (1.19)	0.05	0.12	0.65	0.0035	**0.0257**
N2,N2-dimethylguanosine	Nucleotide	Purine Metabolism, Guanine containing	1.95 (0.8)	1.68 (0.79)	0.0312	2.17	1.85	0.0045	**0.0278**
beta-hydroxyisovalerate	Amino Acid	Leucine, Isoleucine and Valine Metabolism	0.47 (1.29)	-0.07 (1.11)	0.0065	0.33	-0.12	0.009	**0.0478**
sphingomyelin (d18:1/21:0, d17:1/22:0, d16:1/23:0)*	Lipid	Sphingomyelins	-0.75 (0.79)	-0.39 (0.88)	0.0277	-1.04	-0.65	0.0122	0.05
cholesterol	Lipid	Sterol	-0.19 (1.19)	0.24 (1.14)	0.013	-0.45	0.00	0.0133	0.05
1-palmitoyl-2-stearoyl-GPC (16:0/18:0)	Lipid	Phosphatidylcholine (PC)	0.14 (0.89)	0.57 (1.12)	0.0129	0.45	0.03	0.0138	0.05
1,2-dipalmitoyl-GPC (16:0/16:0)	Lipid	Phosphatidylcholine (PC)	-0.16 (1.01)	0.27 (1.17)	0.0236	0.87	0.53	0.0162	0.05
1-(1-enyl-palmitoyl)-2-arachidonoyl-GPE (P-16:0/20:4)*	Lipid	Plasmalogen	-0.08 (1.09)	0.3 (1.05)	0.0282	-0.02	-0.42	0.0379	0.12
1-(1-enyl-palmitoyl)-GPE (P-16:0)*	Lipid	Lysoplasmalogen	-0.18 (1.04)	0.22 (0.97)	0.0219	-0.03	-0.38	0.06	0.18
1-(1-enyl-stearoyl)-2-arachidonoyl-GPE (P-18:0/20:4)*	Lipid	Plasmalogen	-0.21 (0.88)	0.18 (0.94)	0.0104	-0.17	-0.46	0.07	0.19
N-acetyltryptophan	Amino Acid	Tryptophan Metabolism	0.72 (1.11)	0.16 (1.06)	0.004	0.59	0.32	0.08	0.19
N-acetylneuraminate	Carbohydrate	Aminosugar Metabolism	0.65 (0.82)	0.91 (0.79)	0.0451	0.90	0.66	0.08	0.19
1-(1-enyl-palmitoyl)-2-oleoyl-GPE (P-16:0/18:1)*	Lipid	Plasmalogen	-0.01 (1.05)	0.29 (1.01)	0.0289	-0.01	-0.37	0.09	0.19
behenoyl sphingomyelin (d18:1/22:0)*	Lipid	Sphingomyelins	-0.6 (0.67)	-0.38 (0.76)	0.05	-0.65	-0.45	0.09	0.19
thyroxine	Amino Acid	Tyrosine Metabolism	-0.33 (1.17)	0.1 (1.32)	0.0416	-0.10	0.17	0.11	0.19

cysteine-glutathione disulfide	Amino Acid	Glutathione Metabolism	0.11 (1.52)	0.91 (1.45)	0.0026	-0.10	0.37	0.11	0.19
alpha-ketoglutarate	Energy	TCA Cycle	1.16 (1.07)	0.48 (1.37)	0.0038	1.01	1.37	0.11	0.19
1-(1-enyl-oleoyl)-GPE (P-18:1)*	Lipid	Lysoplasmalogen	-0.13 (0.95)	0.26 (0.89)	0.009	0.05	-0.21	0.13	0.22
5,6-dihydrothymine	Nucleotide	Pyrimidine Metabolism, Thymine containing	0.95 (1.04)	0.62 (1.21)	0.042	1.52	1.29	0.14	0.23
1-(1-enyl-stearoyl)-GPE (P-18:0)*	Lipid	Lysoplasmalogen	-0.45 (1.07)	0.06 (0.96)	0.003	-0.19	-0.45	0.16	0.24
N-acetylphenylalanine	Amino Acid	Phenylalanine Metabolism	1.04 (0.99)	0.73 (1.05)	0.0242	1.17	1.02	0.17	0.24
palmitoyl dihydrosphingomyelin (d18:0/16:0)*	Lipid	Dihydrosphingomyelins	-0.61 (0.98)	-0.24 (1.09)	0.0115	-0.49	-0.28	0.17	0.24
cysteine s-sulfate	Amino Acid	Methionine, Cysteine, SAM and Taurine Metabolism	0.44 (1.25)	0.1 (1.2)	0.0409	-1.16	-0.89	0.18	0.25
palmitoyl sphingomyelin (d18:1/16:0)	Lipid	Sphingomyelins	-0.3 (1.04)	0 (1.06)	0.0493	-0.17	-0.01	0.29	0.38
glucuronate	Carbohydrate	Aminosugar Metabolism	0.51 (0.96)	0.07 (1.06)	0.0106	0.66	0.51	0.30	0.38
1-methylnicotinamide	Cofactors and Vitamins	Nicotinate and Nicotinamide Metabolism	-0.4 (0.9)	0.07 (0.82)	0.0016	-0.03	-0.20	0.33	0.40
glycerophosphoethanolamine	Lipid	Phospholipid Metabolism	0.07 (1)	0.39 (1.01)	0.0432	0.08	-0.08	0.36	0.43
imidazole propionate	Amino Acid	Histidine Metabolism	1.03 (0.92)	0.2 (1.58)	0.0007	0.49	0.64	0.37	0.43
glycoursodeoxycholate	Lipid	Secondary Bile Acid Metabolism	0.28 (1.15)	-0.27 (1.15)	0.0077	0.09	0.25	0.40	0.44
cysteine	Amino Acid	Methionine, Cysteine, SAM and Taurine Metabolism	-0.56 (1.36)	-0.13 (1.35)	0.0151	-1.15	-1.29	0.58	0.64
N-acetylglutamate	Amino Acid	Glutamate Metabolism	0.52 (0.52)	0.31 (0.52)	0.0242	0.58	0.54	0.61	0.65
1-stearoyl-GPE (18:0)	Lipid	Lysophospholipid	-0.33 (0.94)	-0.03 (0.92)	0.0405	-0.26	-0.23	0.82	0.84
2-hydroxy-3-methylvalerate	Amino Acid	Leucine, Isoleucine and Valine Metabolism	0.52 (1.05)	0.07 (1.32)	0.0493	0.15	0.16	0.96	0.96

Supplementary Table S5. Changes in metabolomic profile pre- and post-PEA.

Metabolites different between patients with operable CTEPH sampled before surgery and patients sampled post-PEA surgery. Median metabolite levels (z-scores relative to healthy controls) are shown for independent patients before or after PEA surgery, and for a group of 43 patients sampled before and after surgery. Significance shown is for Mann Whitney U tests or paired t-tests, as appropriate. FDR, false discovery rate corrected p-value

Biochemical	Sub-pathway	Super-pathway	Gradients					
			PA-ART.		SVC-PA		PA-SVC	
			FC	FDR p-value	FC	FDR p-value	FC	FDR p-value
Gradients across all sampling sites								
1-methylnicotinamide	Nicotinate and Nicotinamide Metabolism	Cofactors and Vitamins	0.151	6.3668E-06	-0.23	4.8183E-09	-0.079	0.02913395
malate	TCA Cycle	Energy	-0.239	9.3537E-05	-0.264	2.5687E-10	-0.503	5.9814E-12
alpha-ketoglutarate	TCA Cycle	Energy	-0.184	0.00025164	-0.269	8.8517E-08	-0.453	1.8931E-09
arachidonate (20:4n6)	Polyunsaturated Fatty Acid (n3 and n6)	Lipid	-0.241	0.00052156	-0.171	0.00067213	-0.413	1.1959E-08
2-hydroxybutyrate/2-hydroxyisobutyrate	Glutathione Metabolism	Amino Acid	0.073	0.00091228	0.08	0.00298024	0.153	1.6462E-06
glutamate	Glutamate Metabolism	Amino Acid	-0.239	0.00122588	0.998	7.0141E-12	0.759	3.1009E-10
ornithine	Urea cycle; Arginine and Proline Metabolism	Amino Acid	0.142	0.0035656	0.116	0.00596847	0.258	5.1913E-07
glucose	Glycolysis, Gluconeogenesis, and Pyruvate Metabolism	Carbohydrate	-0.115	0.00646371	0.198	3.8473E-06	0.083	0.04600271
eicosapentaenoate (EPA; 20:5n3)	Polyunsaturated Fatty Acid (n3 and n6)	Lipid	-0.127	0.01166334	-0.138	7.3513E-05	-0.265	6.6222E-08
dihomo-linolenate (20:3n3 or n6)	Polyunsaturated Fatty Acid (n3 and n6)	Lipid	-0.151	0.02202636	-0.142	0.00081195	-0.293	1.6462E-06
fumarate	TCA Cycle	Energy	-0.145	0.03812286	-0.346	2.1222E-09	-0.492	5.15E-09
stearidonate (18:4n3)	Polyunsaturated Fatty Acid (n3 and n6)	Lipid	0.078	0.04989586	-0.154	3.2923E-07	-0.076	0.00224968
Gradients between PA-ART and SVC-PA samples								
S-1-pyrroline-5-carboxylate	Glutamate Metabolism	Amino Acid	-0.261	1.6353E-05	0.235	0.0004531		

citrate	TCA Cycle	Energy	0.284	0.00021356	-0.32	1.9078E-07		
hypoxanthine	Purine Metabolism, (Hypo)Xanthine/Inosine containing	Nucleotide	0.246	0.0003471	-0.227	0.00025109		
4-hydroxyphenylpyruvate	Tyrosine Metabolism	Amino Acid	0.238	0.00067592	-0.132	0.01580435		
aspartate	Alanine and Aspartate Metabolism	Amino Acid	-0.318	0.00251419	0.404	2.3314E-05		
methionine sulfoxide	Methionine, Cysteine, SAM and Taurine Metabolism	Amino Acid	-0.143	0.00576202	0.08	0.04780915		
mannose	Fructose, Mannose and Galactose Metabolism	Carbohydrate	-0.089	0.03252006	0.128	0.00201883		
N-acetylmethionine	Methionine, Cysteine, SAM and Taurine Metabolism	Amino Acid	0.087	0.03793341	-0.152	2.6986E-06		
Gradients between PA-ART and ART-SVC samples								
palmitoylcholine	Fatty Acid Metabolism (Acyl Choline)	Lipid	-0.803	2.3129E-09			-0.927	2.1208E-10
glycerophosphorylcholine (GPC)	Phospholipid Metabolism	Lipid	-0.721	6.4598E-09			-0.787	2.0185E-10
sarcosine	Glycine, Serine and Threonine Metabolism	Amino Acid	0.631	1.8199E-08			0.713	1.8931E-09
erythronate*	Aminosugar Metabolism	Carbohydrate	-0.29	3.0496E-08			-0.286	5.15E-09
3-hydroxy-3-methylglutarate	Mevalonate Metabolism	Lipid	-0.378	3.9233E-08			-0.426	2.8368E-10
1-(1-enyl-palmitoyl)-GPE (P-16:0)*	Lysoplasmalogen	Lipid	-0.681	1.3054E-06			-0.604	5.7047E-05
glycerophosphoethanolamine	Phospholipid Metabolism	Lipid	-0.646	1.3149E-06			-0.499	0.00016003
oxalate (ethanedioate)	Ascorbate and Aldarate Metabolism	Cofactors and Vitamins	-0.288	1.3149E-06			-0.282	6.2582E-06
glycerate	Glycolysis, Gluconeogenesis, and Pyruvate Metabolism	Carbohydrate	-0.36	2.363E-06			-0.403	3.3828E-07
1-stearoyl-GPE (18:0)	Lysophospholipid	Lipid	-0.416	6.3668E-06			-0.337	0.0007122

1-linoleoyl-GPC (18:2)	Lysophospholipid	Lipid	-0.396	1.3154E-05			-0.387	6.2582E-06
hypotaurine	Methionine, Cysteine, SAM and Taurine Metabolism	Amino Acid	-0.485	1.5989E-05			-0.557	8.8499E-07
threonate	Ascorbate and Aldarate Metabolism	Cofactors and Vitamins	-0.381	1.5989E-05			-0.34	8.1514E-06
1-(1-enyl-stearoyl)-GPE (P-18:0)*	Lysoplasmalogen	Lipid	-0.569	1.9284E-05			-0.437	0.00166427
1-oleoyl-GPC (18:1)	Lysophospholipid	Lipid	-0.392	2.685E-05			-0.356	3.4486E-05
myo-inositol	Inositol Metabolism	Lipid	-0.318	3.0697E-05			-0.347	4.7834E-07
1-arachidonoyl-GPC (20:4n6)*	Lysophospholipid	Lipid	-0.491	3.4957E-05			-0.501	6.9484E-06
1-linolenoyl-GPC (18:3)*	Lysophospholipid	Lipid	-0.27	3.7291E-05			-0.269	5.1503E-05
1-(1-enyl-oleoyl)-GPE (P-18:1)*	Lysoplasmalogen	Lipid	-0.578	4.0065E-05			-0.529	0.00077158
glucuronate	Aminosugar Metabolism	Carbohydrate	-0.279	8.7596E-05			-0.324	6.1505E-06
cysteine s-sulfate	Methionine, Cysteine, SAM and Taurine Metabolism	Amino Acid	0.412	9.3537E-05			0.428	7.5481E-05
phenol sulfate	Tyrosine Metabolism	Amino Acid	0.058	9.6634E-05			0.071	4.0266E-05
1-stearoyl-GPI (18:0)	Lysophospholipid	Lipid	-0.546	0.00014489			-0.54	3.11E-05
3-methylhistidine	Histidine Metabolism	Amino Acid	0.106	0.0001818			0.095	9.5465E-05
taurine	Methionine, Cysteine, SAM and Taurine Metabolism	Amino Acid	-0.527	0.00021015			-0.394	0.01136516
1-palmitoyl-2-palmitoleoyl-GPC (16:0/16:1)*	Phosphatidylcholine (PC)	Lipid	0.134	0.00036363			0.128	6.2582E-06
arabonate/xylonate	Pentose Metabolism	Carbohydrate	-0.28	0.00039401			-0.333	6.6796E-07
1-palmitoleoyl-GPC (16:1)*	Lysophospholipid	Lipid	-0.23	0.00039401			-0.168	0.00334584
1-arachidonoyl-GPI (20:4)*	Lysophospholipid	Lipid	-0.457	0.00041187			-0.519	3.826E-06
phosphoethanolamine	Phospholipid Metabolism	Lipid	-0.469	0.00067592			-0.322	0.02584305
cysteine-glutathione disulfide	Glutathione Metabolism	Amino Acid	0.211	0.00067592			0.157	0.02344767

1-stearoyl-GPC (18:0)	Lysophospholipid	Lipid	-0.269	0.00087333			-0.261	7.5481E-05
adenosine 5'-monophosphate (AMP)	Purine Metabolism, Adenine containing	Nucleotide	-0.301	0.0016345			-0.4	0.00024698
ribonate	Pentose Metabolism	Carbohydrate	-0.254	0.00171284			-0.195	0.01520143
2-hydroxypalmitate	Fatty Acid, Monohydroxy	Lipid	-0.304	0.00174563			-0.414	6.2582E-06
N-acetylneuraminate	Aminosugar Metabolism	Carbohydrate	-0.297	0.0019371			-0.233	0.03142122
1-oleoyl-GPE (18:1)	Lysophospholipid	Lipid	-0.123	0.00251419			-0.085	0.03894162
1-(1-enyl-palmitoyl)-2-arachidonoyl-GPE (P-16:0/20:4)*	Plasmalogen	Lipid	-0.293	0.00282943			-0.212	0.03515882
N-acetylvaline	Leucine, Isoleucine and Valine Metabolism	Amino Acid	0.118	0.00282943			0.052	0.02630446
1-linoleoyl-GPI (18:2)*	Lysophospholipid	Lipid	-0.325	0.00293949			-0.354	6.3924E-05
allantoin	Purine Metabolism, (Hypo)Xanthine/Inosine containing	Nucleotide	-0.137	0.00313861			-0.117	0.00162672
3-carboxy-4-methyl-5-propyl-2-furanpropanoate (CMPF)	Fatty Acid, Dicarboxylate	Lipid	0.046	0.00348162			0.067	0.00026166
1-myristoyl-2-palmitoyl-GPC (14:0/16:0)	Phosphatidylcholine (PC)	Lipid	0.101	0.00646447			0.129	0.00018146
ethylmalonate	Leucine, Isoleucine and Valine Metabolism	Amino Acid	-0.08	0.00737992			-0.092	0.00021416
asparagine	Alanine and Aspartate Metabolism	Amino Acid	-0.139	0.00751376			-0.216	2.7952E-05
mannitol/sorbitol	Fructose, Mannose and Galactose Metabolism	Carbohydrate	-0.08	0.00767081			-0.058	0.00654856
arabitol/xylitol	Pentose Metabolism	Carbohydrate	-0.1	0.00932002			-0.092	0.00588643
trigonelline (N'-methylnicotinate)	Nicotinate and Nicotinamide Metabolism	Cofactors and Vitamins	0.054	0.01432868			0.049	0.0056586
biliverdin	Hemoglobin and Porphyrin Metabolism	Cofactors and Vitamins	0.124	0.01824202			0.143	0.00642229
1-palmitoyl-GPC (16:0)	Lysophospholipid	Lipid	-0.236	0.02202636			-0.189	0.03328623

1-stearoyl-2-arachidonoyl-GPE (18:0/20:4)	Phosphatidylethanolamine (PE)	Lipid	-0.18	0.02202636			-0.149	0.02584305
1-palmitoyl-2-linoleoyl-GPC (16:0/18:2)	Phosphatidylcholine (PC)	Lipid	0.132	0.02921371			0.214	0.00057078
N1-Methyl-2-pyridone-5-carboxamide	Nicotinate and Nicotinamide Metabolism	Cofactors and Vitamins	0.059	0.02964567			0.056	0.01237472
quinolinate	Nicotinate and Nicotinamide Metabolism	Cofactors and Vitamins	-0.087	0.03223069			-0.091	0.0342017
16a-hydroxy DHEA 3-sulfate	Androgenic Steroids	Lipid	0.042	0.03252006			0.05	0.0097586
1-stearoyl-2-linoleoyl-GPC (18:0/18:2)*	Phosphatidylcholine (PC)	Lipid	0.13	0.03567173			0.15	0.01612179
3-ureidopropionate	Pyrimidine Metabolism, Uracil containing	Nucleotide	-0.18	0.03573385			-0.181	0.018683
1,5-anhydroglucitol (1,5-AG)	Glycolysis, Gluconeogenesis, and Pyruvate Metabolism	Carbohydrate	0.062	0.03793341			0.103	0.00018146
pyridoxate	Vitamin B6 Metabolism	Cofactors and Vitamins	0.069	0.03812286			0.092	0.00247425
1-palmitoyl-2-arachidonoyl-GPI (16:0/20:4)*	Phosphatidylinositol (PI)	Lipid	0.109	0.04000976			0.119	0.0216564
1-stearoyl-2-docosahexaenoyl-GPE (18:0/22:6)*	Phosphatidylethanolamine (PE)	Lipid	0.108	0.04170345			0.107	0.00095383
1-(1-enyl-palmitoyl)-GPC (P-16:0)*	Lysoplasmalogen	Lipid	-0.173	0.04611967			-0.237	0.02347027
phenyllactate (PLA)	Phenylalanine Metabolism	Amino Acid	0.09	0.04664857			0.081	0.02263569
Gradients between SVC-PA and ART-SVC samples								
5,6-dihydrothymine	Pyrimidine Metabolism, Thymine containing	Nucleotide			-0.702	6.1669E-11	-0.548	3.5702E-09
myristoleate (14:1n5)	Long Chain Fatty Acid	Lipid			-0.215	2.2062E-09	-0.138	9.9553E-06
succinate	TCA Cycle	Energy			-0.561	5.6399E-09	-0.518	4.7923E-06
5-dodecenoate (12:1n7)	Medium Chain Fatty Acid	Lipid			-0.188	1.4414E-08	-0.115	2.7952E-05
glycerol	Glycerolipid Metabolism	Lipid			-0.351	4.492E-08	-0.346	5.2117E-06

orotate	Pyrimidine Metabolism, Orotate containing	Nucleotide			-0.182	6.148E-08	-0.265	2.4564E-09
laurate (12:0)	Medium Chain Fatty Acid	Lipid			-0.18	4.0707E-07	-0.247	2.9391E-09
myristate (14:0)	Long Chain Fatty Acid	Lipid			-0.189	4.6872E-07	-0.123	0.00057078
7-alpha-hydroxy-3-oxo-4-cholestenoate (7-Hoca)	Sterol	Lipid			-0.308	7.0852E-07	-0.284	1.4406E-08
linolenate [alpha or gamma; (18:3n3 or 6)]	Polyunsaturated Fatty Acid (n3 and n6)	Lipid			-0.144	1.61E-06	-0.07	0.00959807
3-hydroxylaurate	Fatty Acid, Monohydroxy	Lipid			-0.174	4.7676E-06	-0.151	6.3924E-05
gamma-glutamylglutamate	Gamma-glutamyl Amino Acid	Peptide			0.406	2.2447E-05	0.536	6.0645E-08
xanthine	Purine Metabolism, (Hypo)Xanthine/Inosine containing	Nucleotide			-0.12	2.7189E-05	-0.096	7.5074E-05
3-hydroxybutyrate (BHBA)	Ketone Bodies	Lipid			0.128	3.1815E-05	0.191	1.7422E-06
creatine	Creatine Metabolism	Amino Acid			0.135	5.4254E-05	0.154	7.5074E-05
choline	Phospholipid Metabolism	Lipid			-0.182	7.0016E-05	-0.127	0.00450938
caprate (10:0)	Medium Chain Fatty Acid	Lipid			-0.114	7.1324E-05	-0.106	0.00017611
glutamine	Glutamate Metabolism	Amino Acid			-0.241	0.00014192	-0.165	0.0216564
docosapentaenoate (n3 DPA; 22:5n3)	Polyunsaturated Fatty Acid (n3 and n6)	Lipid			-0.151	0.00037289	-0.154	0.00088718
linoleate (18:2n6)	Polyunsaturated Fatty Acid (n3 and n6)	Lipid			-0.128	0.00038275	-0.086	0.01338683
5-oxoproline	Glutathione Metabolism	Amino Acid			-0.204	0.00053368	-0.188	0.0088785
trans-4-hydroxyproline	Urea cycle; Arginine and Proline Metabolism	Amino Acid			-0.11	0.00067213	-0.124	5.7976E-05
citrulline	Urea cycle; Arginine and Proline Metabolism	Amino Acid			0.163	0.00076679	0.151	0.00331986
alanine	Alanine and Aspartate Metabolism	Amino Acid			-0.223	0.0008132	-0.211	0.00196605

docosahexaenoate (DHA; 22:6n3)	Polyunsaturated Fatty Acid (n3 and n6)	Lipid			-0.159	0.00094698	-0.228	6.2582E-06
deoxycarnitine	Carnitine Metabolism	Lipid			-0.12	0.00160152	-0.087	0.02712928
guanidinoacetate	Creatine Metabolism	Amino Acid			-0.118	0.00199572	-0.2	6.5855E-05
caprylate (8:0)	Medium Chain Fatty Acid	Lipid			-0.169	0.0036433	-0.133	0.03840176
serine	Glycine, Serine and Threonine Metabolism	Amino Acid			0.14	0.00935717	0.112	0.00725554
pentadecanoate (15:0)	Long Chain Fatty Acid	Lipid			-0.157	0.01427051	-0.093	0.04481935
3-methyl-2-oxovalerate	Leucine, Isoleucine and Valine Metabolism	Amino Acid			0.143	0.01624892	0.123	0.03395985
indoleacetate	Tryptophan Metabolism	Amino Acid			0.054	0.01785248	0.076	0.00272741
phosphate	Oxidative Phosphorylation	Energy			0.164	0.01927463	0.274	5.7047E-05
13-HODE + 9-HODE	Fatty Acid, Monohydroxy	Lipid			-0.13	0.02857954	-0.184	0.00280084
4-methyl-2-oxopentanoate	Leucine, Isoleucine and Valine Metabolism	Amino Acid			0.081	0.03931529	0.102	0.01237472
Gradients between PA-ART samples								
choline phosphate	Phospholipid Metabolism	Lipid	-0.471	0.0007693				
1-(1-enyl-stearoyl)-2-arachidonoyl-GPE (P-18:0/20:4)*	Plasmalogen	Lipid	-0.294	0.00168983				
nicotinamide	Nicotinate and Nicotinamide Metabolism	Cofactors and Vitamins	-0.394	0.00535394				
homoarginine	Urea cycle; Arginine and Proline Metabolism	Amino Acid	0.092	0.00576202				
1-palmitoyl-GPE (16:0)	Lysophospholipid	Lipid	-0.205	0.00646447				
bilirubin (E,E)*	Hemoglobin and Porphyrin Metabolism	Cofactors and Vitamins	-0.207	0.00978439				
1-(1-enyl-palmitoyl)-2-oleoyl-GPE (P-16:0/18:1)*	Plasmalogen	Lipid	-0.199	0.01436973				
betaine	Glycine, Serine and Threonine Metabolism	Amino Acid	0.089	0.0280844				

1-arachidonoyl-GPE (20:4n6)*	Lysophospholipid	Lipid	-0.123	0.03247541				
Gradients between SVC-PA samples								
lactate	Glycolysis, Gluconeogenesis, and Pyruvate Metabolism	Carbohydrate			-0.215	2.1564E-06		
N-formylmethionine	Methionine, Cysteine, SAM and Taurine Metabolism	Amino Acid			-0.185	6.8059E-06		
7-methylguanine	Purine Metabolism, Guanine containing	Nucleotide			-0.165	0.00016298		
3-(4-hydroxyphenyl)lactate	Tyrosine Metabolism	Amino Acid			-0.124	0.00040297		
N-acetylputrescine	Polyamine Metabolism	Amino Acid			-0.109	0.0004531		
isoleucine	Leucine, Isoleucine and Valine Metabolism	Amino Acid			0.171	0.00144109		
palmitoleate (16:1n7)	Long Chain Fatty Acid	Lipid			-0.116	0.00152404		
10-heptadecenoate (17:1n7)	Long Chain Fatty Acid	Lipid			-0.109	0.00201883		
3-hydroxy-2-ethylpropionate	Leucine, Isoleucine and Valine Metabolism	Amino Acid			-0.201	0.0043224		
3-hydroxydecanoate	Fatty Acid, Monohydroxy	Lipid			-0.091	0.00465296		
trans-urocanate	Histidine Metabolism	Amino Acid			-0.343	0.01140618		
1-palmitoyl-2-stearoyl-GPC (16:0/18:0)	Phosphatidylcholine (PC)	Lipid			0.182	0.01629051		
3-hydroxyoctanoate	Fatty Acid, Monohydroxy	Lipid			-0.097	0.02857954		
Gradients between ART-SVC samples								
leucine	Leucine, Isoleucine and Valine Metabolism	Amino Acid					0.174	0.00028644
1-stearoyl-2-docosahexaenoyl-GPC (18:0/22:6)	Phosphatidylcholine (PC)	Lipid					0.126	0.0003638
sphingomyelin (d18:2/23:0, d18:1/23:1, d17:1/24:1)*	Sphingomyelins	Lipid					0.119	0.0009553
vanillylmandelate (VMA)	Tyrosine Metabolism	Amino Acid					0.118	0.00216943

gamma-glutamyl-epsilon-lysine	Gamma-glutamyl Amino Acid	Peptide					-0.265	0.00228894
1-palmitoyl-2-oleoyl-GPC (16:0/18:1)	Phosphatidylcholine (PC)	Lipid					0.177	0.00241612
imidazole propionate	Histidine Metabolism	Amino Acid					0.129	0.00246402
1-stearoyl-2-oleoyl-GPC (18:0/18:1)	Phosphatidylcholine (PC)	Lipid					0.173	0.00255738
1-linoleoyl-2-linolenoyl-GPC (18:2/18:3)*	Phosphatidylcholine (PC)	Lipid					0.122	0.00267674
gamma-glutamylmethionine	Gamma-glutamyl Amino Acid	Peptide					-0.199	0.00298366
1-myristoyl-2-arachidonoyl-GPC (14:0/20:4)*	Phosphatidylcholine (PC)	Lipid					0.116	0.00331986
1-(1-enyl-stearoyl)-2-linoleoyl-GPE (P-18:0/18:2)*	Plasmalogen	Lipid					0.086	0.00399784
sphingomyelin (d18:1/21:0, d17:1/22:0, d16:1/23:0)*	Sphingomyelins	Lipid					0.085	0.00520102
1-palmitoyl-2-docosahexaenoyl-GPE (16:0/22:6)*	Phosphatidylethanolamine (PE)	Lipid					0.083	0.00586853
1-oleoyl-2-docosahexaenoyl-GPC (18:1/22:6)*	Phosphatidylcholine (PC)	Lipid					0.18	0.00725554
N-acetylserine	Glycine, Serine and Threonine Metabolism	Amino Acid					-0.108	0.00791607
1,2-dilinoleoyl-GPC (18:2/18:2)	Phosphatidylcholine (PC)	Lipid					0.1	0.00828729
phenylpyruvate	Phenylalanine Metabolism	Amino Acid					0.215	0.00918305
glycochenodeoxycholate glucuronide (1)	Primary Bile Acid Metabolism	Lipid					0.066	0.00952848
N-acetyltryptophan	Tryptophan Metabolism	Amino Acid					0.12	0.01174907
androsterone sulfate	Androgenic Steroids	Lipid					0.067	0.01181751
indolepropionate	Tryptophan Metabolism	Amino Acid					0.052	0.01237472
1-stearoyl-2-arachidonoyl-GPC (18:0/20:4)	Phosphatidylcholine (PC)	Lipid					0.145	0.01262466

glycocholenate sulfate*	Secondary Bile Acid Metabolism	Lipid					0.095	0.01447754
1-palmitoyl-2-linoleoyl-GPE (16:0/18:2)	Phosphatidylethanolamine (PE)	Lipid					0.07	0.01520143
sphingomyelin (d18:1/18:1, d18:2/18:0)	Sphingomyelins	Lipid					0.076	0.01520143
sphingomyelin (d18:1/20:1, d18:2/20:0)*	Sphingomyelins	Lipid					0.094	0.01547065
orotidine	Pyrimidine Metabolism, Orotate containing	Nucleotide					-0.06	0.01618316
gamma-glutamylglutamine	Gamma-glutamyl Amino Acid	Peptide					-0.202	0.01651043
sphingomyelin (d18:1/22:1, d18:2/22:0, d16:1/24:1)*	Sphingomyelins	Lipid					0.11	0.01774506
tryptophan	Tryptophan Metabolism	Amino Acid					0.109	0.01784552
1-(1-enyl-palmitoyl)-2-arachidonoyl-GPC (P-16:0/20:4)*	Plasmalogen	Lipid					0.114	0.01790998
2-hydroxystearate	Fatty Acid, Monohydroxy	Lipid					-0.238	0.02142168
N-acetylalanine	Alanine and Aspartate Metabolism	Amino Acid					-0.128	0.0222828
trimethylamine N-oxide	Phospholipid Metabolism	Lipid					0.056	0.02263569
alpha-hydroxyisovalerate	Leucine, Isoleucine and Valine Metabolism	Amino Acid					0.057	0.02375697
1-palmitoyl-2-docosahexaenoyl-GPC (16:0/22:6)	Phosphatidylcholine (PC)	Lipid					0.126	0.02492348
uridine	Pyrimidine Metabolism, Uracil containing	Nucleotide					0.095	0.02766267
N-methylproline	Urea cycle; Arginine and Proline Metabolism	Amino Acid					0.041	0.02913395
1-linoleoyl-2-arachidonoyl-GPC (18:2/20:4n6)*	Phosphatidylcholine (PC)	Lipid					0.099	0.03010652
N-acetylphenylalanine	Phenylalanine Metabolism	Amino Acid					0.092	0.03270972

3-methylglutarylcarnitine (2)	Leucine, Isoleucine and Valine Metabolism	Amino Acid					0.079	0.03413624
sphingomyelin (d18:2/24:1, d18:1/24:2)*	Sphingomyelins	Lipid					0.099	0.0342017
1,2-dipalmitoyl-GPC (16:0/16:0)	Phosphatidylcholine (PC)	Lipid					0.145	0.03528075
phenylacetylglutamine	Acetylated Peptides	Peptide					0.063	0.03894162
2-hydroxy-3-methylvalerate	Leucine, Isoleucine and Valine Metabolism	Amino Acid					0.091	0.04416028
sphingomyelin (d18:1/17:0, d17:1/18:0, d19:1/16:0)	Sphingomyelins	Lipid					0.083	0.04456191
1-(1-enyl-palmitoyl)-2-linoleoyl-GPC (P-16:0/18:2)*	Plasmalogen	Lipid					0.068	0.0462457

Supplementary Table S6. Full list of metabolites with nominally significant tissue gradients

Sub-pathway	Metabolites tested in the pathway	SVC-PA	Sig.	PA-ART	Sig.	ART-SVC	Sig.	SVC-PA only	Sig.	PA-ART only	Sig.	ART-SVC only	Sig.
Pathway showing enrichment													
Polyunsaturated Fatty Acid (n3 and n6)	12	8	0.0002	4	0.31	8	0.0241						
TCA Cycle	6	5	0.0008	4	0.0243	4	0.1						
Medium Chain Fatty Acid	5	4	0.0038			4	0.0431						
Phosphatidylcholine (PC)	17	1	0.33	4	1	15	0.0000	1	0.5			11	0
Nicotinate and Nicotinamide Metabolism	5	1	1	5	0.0005	4	0.0431			1	0.13		
Phospholipid Metabolism	6	1	1	4	0.0243	5	0.0166			1	0.15	1	0.57
Lysophospholipid	23			14	0.0000	12	0.07			2	0.12		
Lysoplasmalogen	4			4	0.0024	4	0.0116						
Aminosugar Metabolism	4			3	0.0363	3	0.11						
Pentose Metabolism	4			3	0.0363	3	0.11						
Plasmalogen	11			3	0.71	4	0.76			2	0.0322	3	0.16
Secondary Bile Acid Metabolism	15					1	0.0261					1	0.7
Sphingomyelins	16					7	0.42					7	0.0019
Pathways not showing enrichment													
Long Chain Fatty Acid	14	5	0.08			3	0.41	2	0.1				
Glutamate Metabolism	9	3	0.2	2	1	2	0.72						
Purine Metabolism, (Hypo)Xanthine/Inosine containing	6	2	0.28	2	0.62	2	1						
Tryptophan Metabolism	14	1	0.48			4	1					3	0.41
Fructose, Mannose and Galactose Metabolism	4	1	0.54	2	0.22	1	1						
Sterol	4	1	0.54			1	1						
Histidine Metabolism	11	1	0.7	1	0.47	2	0.35	1	0.36			1	1

Leucine, Isoleucine and Valine Metabolism	21	4	0.77	2	0.19	8	0.64	2	0.19			4	0.5
Urea cycle; Arginine and Proline Metabolism	16	3	1	2	0.54	4	0.6			1	0.37	1	0.71
Methionine, Cysteine, SAM and Taurine Metabolism	17	3	1	5	0.55	3	0.2	1	0.5				
Tyrosine Metabolism	14	2	1	2	0.74	2	0.16	1	0.43			1	1
Glycine, Serine and Threonine Metabolism	10	1	1	2	1	3	1			1	0.25	1	1
Polyamine Metabolism	5	1	1					1	0.18				
Androgenic Steroids	11			1	0.47	2	0.35					1	1
Hemoglobin and Porphyrin Metabolism	7			2	0.66	1	0.43			1	0.18		
Fatty Acid, Dicarboxylate	4			1	1	1	1						
Phosphatidylinositol (PI)	5			1	1	1	1						
Purine Metabolism, Adenine containing	4			1	1	1	1						
Primary Bile Acid Metabolism	9					1	0.28					1	1
Acetylated Peptides	4					1	1					1	0.43

Supplementary Table S7. Enrichment analysis of all pathways. Enrichment was first tested for metabolites with gradients in each analysis, irrespective of overlap between analyses, and then in those metabolites specifically with gradients in only one of the three analyses, SVC-PA, PA-ART or ART-SVC.

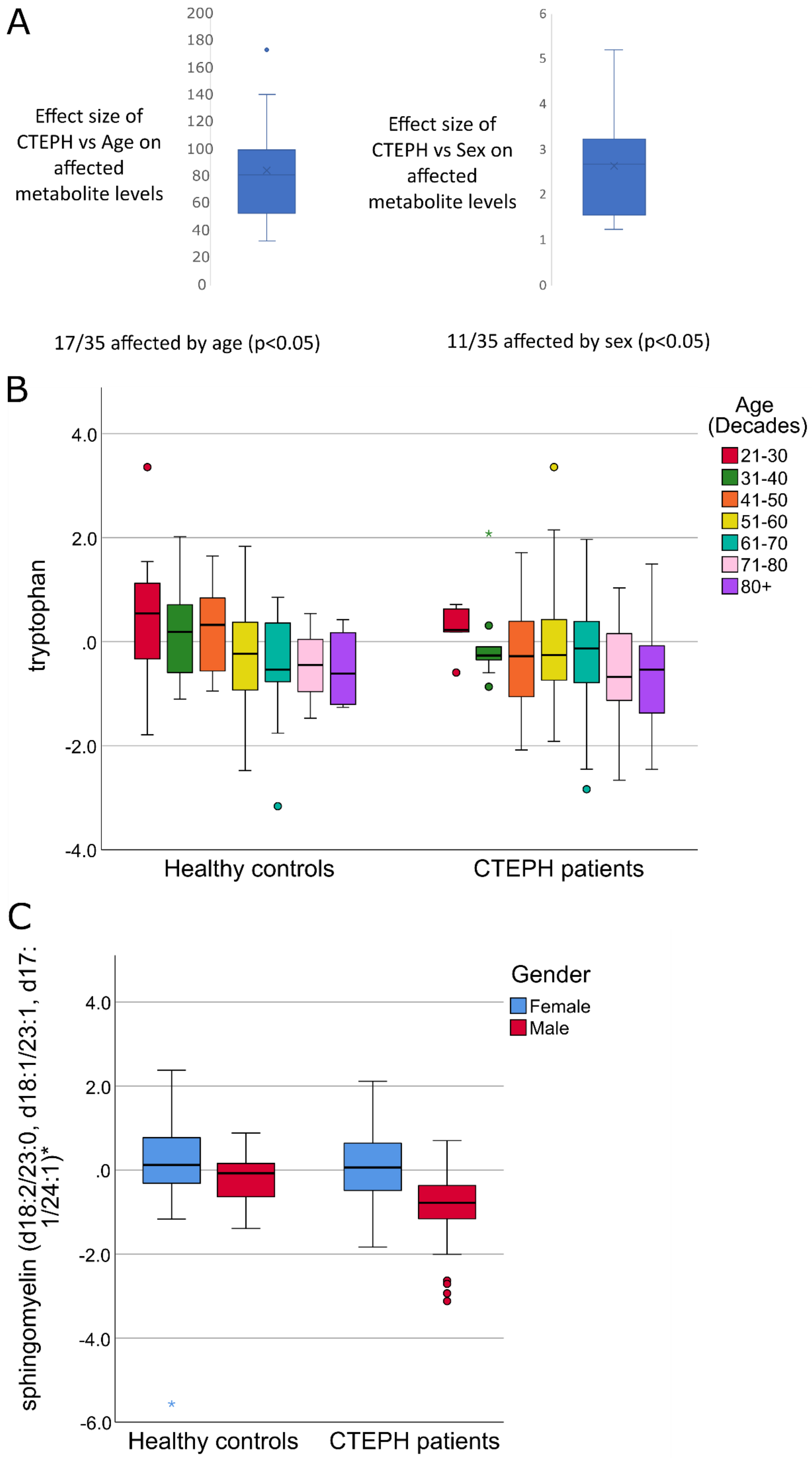
A
Effect size of CTEPH vs Age on affected metabolite levels
200
180
160
140
120
100
80
60
40
20
0
Effect size of CTEPH vs Sex on affected metabolite levels
6
5
4
3
2
1
0
17/35 affected by age (p<0.05)
11/35 affected by sex (p<0.05)
B
tryptophan
4.0
2.0
.0
-2.0
-4.0
Healthy controls
CTEPH patients
Age (Decades)
21-30
31-40
41-50
51-60
61-70
71-80
80+
C
sphingomyelin (d18:2/23:0, d18:1/23:1, d17:1/24:1)*
4.0
2.0
.0
-2.0
-4.0
-6.0
Healthy controls
CTEPH patients
Gender
Female
Male

Supplementary Figure S1 – Effect of age and gender on metabolites associated with CTEPH. A. Ratio of effect sizes of CTEPH (versus healthy controls) compared to age and gender for metabolites significantly affected by age or gender, indicating generally larger (>1 ratio) effect of CTEPH status. B. Boxplot of CTEPH-associated metabolite most affected by age, tryptophan, in healthy controls and CTEPH patients divided by age in decades. C. Boxplot of CTEPH-associated metabolite most affected by gender, sphingomyelin (d18:2/23:0, d18.1/23:1, d17:1/24:1)*, in healthy controls and CTEPH patients divided by self-reported gender.

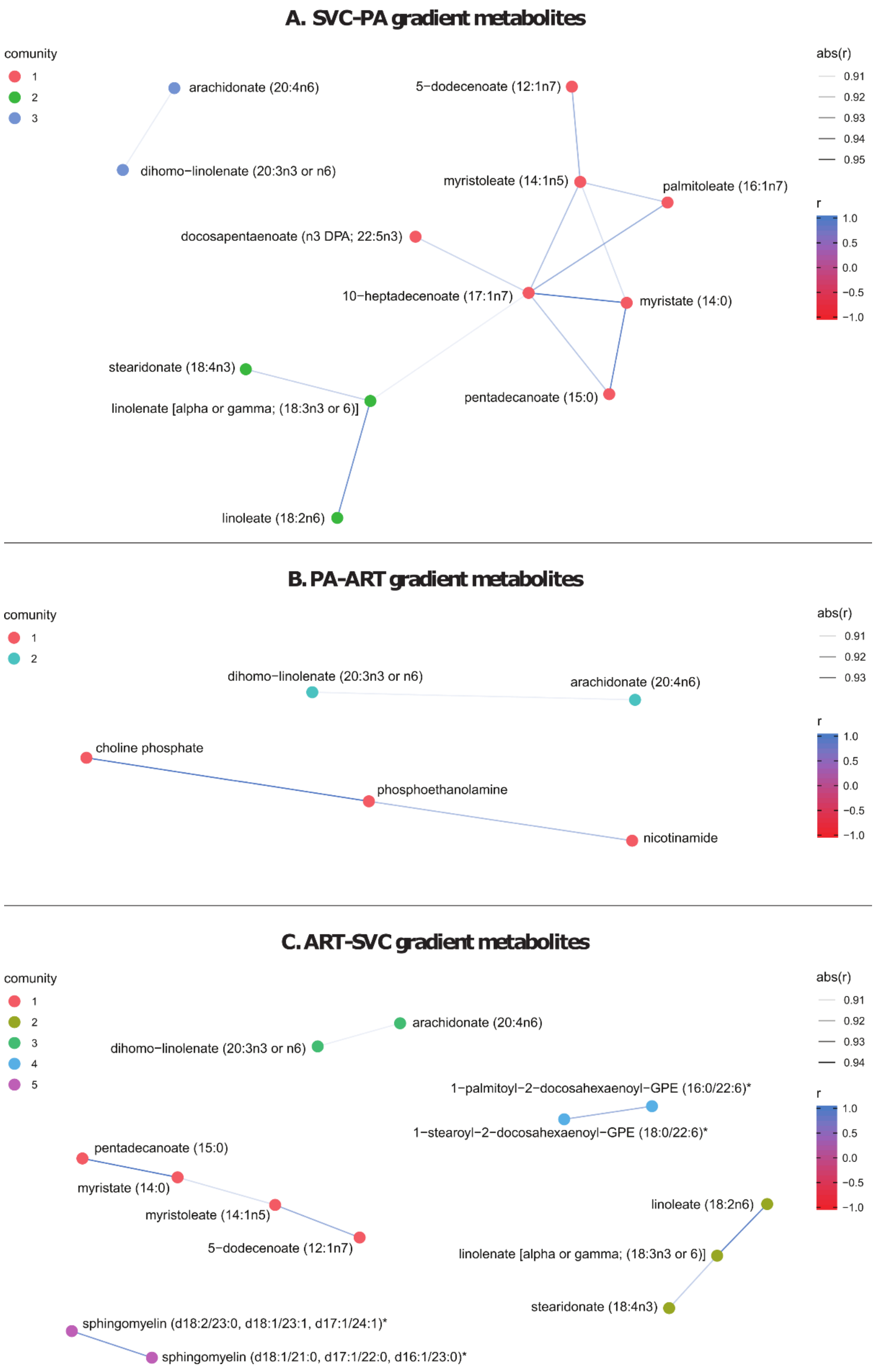

Supplementary Figure S2. Relevance network analysis. Network of highly correlated (r>0.9) metabolites showing SVC-PA (A), PA-ART (B) or ART-SVC (C) gradients.

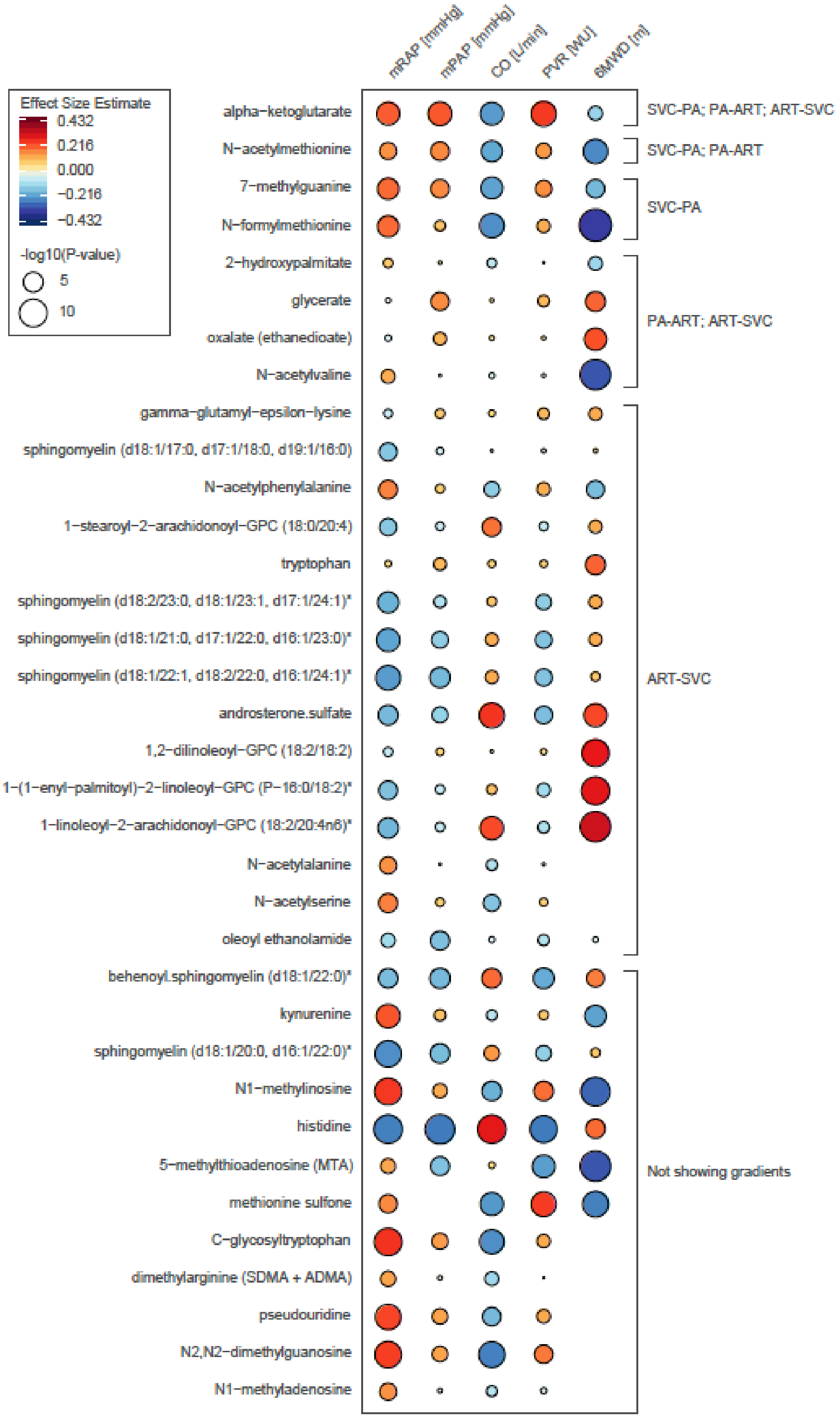

Supplementary Figure S3. Associations between clinical variables and plasma metabolites that distinguish CTEPH from healthy and disease controls. Directionality and magnitude of estimates are presented by colour fill scale (red for positive and blue for negative directionality), corresponding p-values are represented by circle size.

Supplementary References

1. Rosenkranz S, Preston IR. Right heart catheterisation: best practice and pitfalls in pulmonary hypertension. Eur Respir Rev. 2015;24(138):642-52.

2. Shenoy V, Anton JM, Collard CD, Youngblood SC. Pulmonary thromboendarterectomy for chronic thromboembolic pulmonary hypertension. Anesthesiology. 2014;120(5):1255-61.

3. Jamieson SW, Kapelanski DP, Sakakibara N, Manecke GR, Thistlethwaite PA, Kerr KM, et al. Pulmonary endarterectomy: experience and lessons learned in 1,500 cases. Ann Thorac Surg. 2003;76(5):1457-62; discussion 62-4.
