# Supplementary material for: Plasma metabolomics exhibit response to therapy in chronic thromboembolic pulmonary hypertension
Source: Eur Respir J. 2021 Apr 1;57(4):2003201. doi: 10.1183/13993003.03201-2020 (PMC8012591; doi:10.1183/13993003.03201-2020)
Supplement: Supplementary file 2 [file ERJ-03201-2020.Shareable.pdf]

SHAREABLE PDF

# Plasma metabolomics exhibit response to therapy in chronic thromboembolic pulmonary hypertension

Emilia M. Swietlik[1,2], Pavandeep Ghataorhe[3], Kasia I. Zalewska[2,4], John Wharton[3], Luke S. Howard[5], Dolores Taboada[2], John E. Cannon[2], UK National Cohort Study of PAH, Nicholas W. Morrell[1], Martin R. Wilkins[3], Mark Toshner[1,2], Joanna Pepke-Zaba[1,2] and Christopher J. Rhodes[3]

**Affiliations**: [1]Dept of Medicine, University of Cambridge, Cambridge, UK. [2]National Pulmonary Hypertension Service, Royal Papworth Hospital, Cambridge, UK. [3]National Heart and Lung Institute, Medicine, Imperial College London, London, UK. [4]Respiratory Unit, University Hospital Llandough, Cardiff, UK. [5]National Pulmonary Hypertension Service, Imperial College Healthcare NHS Trust and NHLI, Imperial College, Hammersmith Hospital, London, UK.

**Correspondence**: Christopher J. Rhodes, National Heart and Lung Institute, Medicine, Imperial College London, Hammersmith Campus, London W12 0NN, UK. E-mail: crhodes@imperial.ac.uk

@ERSpublications
**Metabolic profiles can distinguish CTEPH from controls and disease comparators, but most metabolic changes are common to both CTEPH and IPAH. Cardiopulmonary tissue metabolism is relevant to PH and metabolites do respond to PEA surgery.** https://bit.ly/368ogtG

**Cite this article as:** Swietlik EM, Ghataorhe P, Zalewska KI, *et al.* Plasma metabolomics exhibit response to therapy in chronic thromboembolic pulmonary hypertension. *Eur Respir J* 2021; 57: 2003201 [https://doi.org/10.1183/13993003.03201-2020].

This single-page version can be shared freely online.

ABSTRACT Pulmonary hypertension is a condition with limited effective treatment options. Chronic thromboembolic pulmonary hypertension (CTEPH) is a notable exception, with pulmonary endarterectomy (PEA) often proving curative. This study investigated the plasma metabolome of CTEPH patients, estimated reversibility to an effective treatment and explored the source of metabolic perturbations.

We performed untargeted analysis of plasma metabolites in CTEPH patients compared to healthy controls and disease comparators. Changes in metabolic profile were evaluated in response to PEA. A subset of patients were sampled at three anatomical locations and plasma metabolite gradients calculated.

We defined and validated altered plasma metabolite profiles in patients with CTEPH. 12 metabolites were confirmed by receiver operating characteristic analysis to distinguish CTEPH and both healthy (area under the curve (AUC) 0.64–0.94, all $p<2\times10^{-5}$) and disease controls (AUC 0.58–0.77, all $p<0.05$). Many of the metabolic changes were notably similar to those observed in idiopathic pulmonary arterial hypertension (IPAH). Only five metabolites (5-methylthioadenosine, N1-methyladenosine, N1-methylinosine, 7-methylguanine, N-formylmethionine) distinguished CTEPH from chronic thromboembolic disease or IPAH. Significant corrections (15–100% of perturbation) in response to PEA were observed in some, but not all metabolites. Anatomical sampling identified 188 plasma metabolites, with significant gradients in tryptophan, sphingomyelin, methionine and Krebs cycle metabolites. In addition, metabolites associated with CTEPH and gradients showed significant associations with clinical measures of disease severity.

We identified a specific metabolic profile that distinguishes CTEPH from controls and disease comparators, despite the observation that most metabolic changes were common to both CTEPH and IPAH patients. Plasma metabolite gradients implicate cardiopulmonary tissue metabolism of metabolites associated with pulmonary hypertension and metabolites that respond to PEA surgery could be a suitable noninvasive marker for evaluating future targeted therapeutic interventions.

Copyright ©ERS 2021. This version is distributed under the terms of the Creative Commons Attribution Licence 4.0.
